# From Biomass to Efficient Lipid Recovery: Choline-Based Ionic Liquids and Microwave Extraction of *Chlorella vulgaris*

**DOI:** 10.3390/molecules30234611

**Published:** 2025-12-01

**Authors:** Daniela A. S. Agostinho, Andreia F. M. Santos, José M. S. S. Esperança, Patrícia M. Reis, Ana Rita C. Duarte, Márcia G. Ventura

**Affiliations:** 1 Laboratório Associado para a Química Verde e Tecnologias Sustentáveis-Rede de Química e Tecnologia (LAQV-REQUIMTE), Departamento de Química, NOVA School of Science and Technology, Universidade NOVA de Lisboa, Campus da Caparica, 2829-516 Caparica, Portugal; d.agostinho@campus.fct.unl.pt (D.A.S.A.); jmesp@fct.unl.pt (J.M.S.S.E.); preis@fct.unl.pt (P.M.R.); 2 Escola Superior de Tecnologia do Barreiro, Instituto Politécnico de Setúbal (ESTB/IPS), 2839-001 Lavradio, Portugal

**Keywords:** *Chlorella vulgaris*, ionic liquid, microwave-assisted extraction, lipids, FAMEs

## Abstract

The sustainable extraction of microalgal lipids represents a critical step toward the valorization of biomass for nutraceutical, pharmaceutical, and biofuel applications. In this study, a microwave-assisted extraction approach using a biocompatible ionic liquid (IL), [N_1 1 2OH 2OH_][C_6_H_11_O_2_], was investigated for lipid recovery from *Chlorella vulgaris*. Conventional methods (Soxhlet, Folch, and Bligh & Dyer) were first evaluated for benchmarking. Optimization of microwave power, extraction time, and algae-to-IL mass ratio demonstrated that a 1:8 (*m*/*m*) ratio under 5 min and 750 W microwave pretreatment achieved the highest lipid yield, with 10.61 ± 0.39% lipids recovered from the supernatant alone. Subsequent extraction of the pretreated biomass using an environmentally benign solvent mixture (ethyl acetate/ethanol, 1:1 *v*/*v*) raised the total lipid recovery to 14.29 ± 0.75%, surpassing Soxhlet extraction with chloroform/methanol (13.04 ± 0.16%). Importantly, the IL was efficiently recovered (≈85%) and reused without significant loss of performance or structural integrity, as confirmed by NMR, DSC, and FTIR analyses. The combined process yielded up to 42.56 ± 0.64 mg FAMEs/g algae, comparable to conventional Soxhlet extraction but with superior environmental compatibility. The relative distribution of FAMEs (in weight percent, wt%) was as follows: C16:2 trans 6.05%, C16:3 trans 13.99%, C16:1 cis 1.85%, C16:1 trans 0.82%, C16:0 16.72%, C18:2 cis 13.74%, C18:3 trans + C18:1 cis + C18:2 trans 26.91%, C18:1 trans 1.67% and C18:0 3.61%. These findings demonstrate that microwave-assisted extraction with choline-based ILs offers an efficient, recyclable, and greener alternative for lipid and fatty acid recovery from microalgae.

## 1. Introduction

The growing demand for health-promoting and environmentally friendly products has intensified interest in natural bioactive compounds with antioxidant, antiviral, antibacterial, anticancer, anticoagulant, anti-inflammatory, antihypertensive properties and other beneficial activities. These compounds, commonly extracted from plants, algae, and fish must be consciously explored and sustainably utilized [1,2,3,4,5].

Microalgae are promising renewable feedstocks, capable of producing lipids, proteins, polysaccharides, pigments, and other valuable molecules for applications in food, feed, cosmetics, pharmaceuticals, and biofuels [2,6,7,8]. Among them, *Chlorella vulgaris* (*C. vulgaris*) stands out for its rigid cell wall, high chlorophyll, protein, vitamin and mineral content, and its ability to accumulate lipids and carotenoids under specific conditions [1,4,9]. Rich in fatty acids, such as palmitic (C16:0), oleic (C18:1), linoleic (C18:2) and linolenic (C18:3) acids, *C. vulgaris* represents a potential alternative source for marine lipids [2,9].

Lipids are generally classified as reserve (neutral or non-polar) lipids, being stored as lipid droplets in the chloroplast and cytoplasm and membrane (polar or complex) lipids that are present in the cell wall and organelle membranes [1,2,10,11]. Among the different types of lipids are fatty acids, which differ in chain length and degree of unsaturation, ranging from saturated to monounsaturated and polyunsaturated fatty acids [12]. While, microalgal lipids and fatty acids exhibit diverse bioactivities, including antioxidant, antibacterial, antifungal, anti-inflammatory, antihypertensive, and immunomodulatory effects, also contributing to cardiovascular, neurological, and metabolic health [2,13,14,15,16,17,18,19,20,21,22,23,24,25,26,27,28,29], polyunsaturated fatty acids from microalgae have high nutritional value and are used in health products, functional foods, and dietary supplements. [30,31]. Microalgal oil is therefore considered a promising alternative to fish oil [2]. Essential omega-3 (ω-3, e.g., eicosapentaenoic acid (EPA), docosahexaenoic acid (DHA), and α-linolenic acid) and omega-6 (ω-6, e.g., linoleic acid, γ-linolenic acid, and arachidonic acid) are crucial for human health; fatty acids must be balanced in the diet, with a preference for higher omega-3 intake [32].

Over time, researchers have developed extraction methods for microalgal oils that reduce solvent and energy consumption and minimize environmental impact and cost, while improving yields and preserving lipid quality [33]. Microalgal cell walls are composed of complex polysaccharide (e.g., cellulose) and protein matrices, which makes them resistant to mechanical disruption and limits solvent penetration [34,35]. Having this in mind, efficient extraction requires suitable pretreatments to disrupt the cell wall and solvents with a polarity compatible with the target compounds [2,33].

Traditionally, lipid extraction from microalgae has relied on the Soxhlet [36], Folch [37], and Bligh & Dyer [38] methods, all using organic solvents. The Soxhlet method employs hexane at high temperatures, leading to high energy consumption [35]. In contrast, the Folch et al. method operates at room temperature with a chloroform–methanol mixture, while the Bligh & Dyer method, a modification of Folch, extracts total lipids using chloroform, methanol, and water. Both Folch and Bligh & Dyer methods efficiently recover neutral and polar lipids [10]. Hexane is preferred industrially due to its low polarity, which minimizes co-extraction of polar contaminants and its recoverability through fractional distillation. However, hexane is unable to extract lipids stored in lipid droplets, since it cannot penetrate the polar phospholipid membrane nor break protein bonds, resulting in inadequate lipid extraction [7,33,34].

Organic solvents are highly volatile, raising concerns for both human health and the environment. Chloroform is metabolized into reactive compounds like phosgene, causing liver and kidney toxicity, central nervous system damage, and potential carcinogenicity while persisting in air, water, and soil [39,40,41]. Methanol is converted to formaldehyde and formic acid, leading to metabolic acidosis, neurotoxicity, and severe retinal damage [40,42]. Hexane’s metabolite 2,5-hexanedione induces peripheral neuropathy, reproductive toxicity, and multi-organ oxidative damage [41,43]. Moreover, solvent-based extraction is often time-consuming, requires large solvent volumes, and frequently demands heating to high temperatures, which increases costs, energy consumption, and environmental impact [10,44]. Greener and more efficient extraction strategies are therefore needed, along with the recovery and reuse of solvents when replacement is not feasible. Pretreatment of biomass to weaken the robust microalgal cell walls is commonly employed to enhance lipid release under milder conditions [10,45]. The use of less harmful alternative solvents is crucial to reduce disposal costs, legal liabilities, and environmental impact [44,46]. In this context, “green solvents”, such as ionic liquids (ILs), have emerged as promising alternatives, demonstrating the potential to replace conventional solvents like chloroform, methanol, and hexane for extracting lipids and other bioactive compounds from microalgae [6,7,34,35,44,45,46,47,48,49,50,51,52,53,54,55,56].

ILs are organic salts with low melting point, typically below 100 °C, composed of organic cations and organic or inorganic anions. They offer unique properties, including high thermal and electrochemical stability, low vapor pressure, non-flammability, high conductivity, wide miscibility, excellent microwave absorption, and the ability to be recycled and reused. Many ILs also exhibit relatively low toxicity and can be synthesized in a cost-effective manner [2,6,7,34,46,53]. Their structural flexibility allows them to be “designer solvents,” with tunable melting points, viscosity, polarity, and miscibility to suit specific applications [8,44]. However, not all ILs are truly “green”: some are toxic, poorly biodegradable, or rely on fossil-derived reagents [2,10]. The environmental impact of ILs can nonetheless be minimized by the above-mentioned careful molecular design and efficient recycling. ILs based on imidazolium and pyridinium cations are generally toxic and poorly biodegradable, whereas choline-based ILs are safer and more sustainable [57,58,59]. ILs containing hydrolyzable esters, oxidizable alcohol groups, or carboxylic acids may exhibit enhanced biodegradability due to these functional moieties [10]. Nevertheless, the ultimate fate of the degradation products, including the central cation and anion, remains uncertain and may vary on a case-by-case basis. Long alkyl chains in the cation are generally associated with increased toxicity. Therefore, preparing ILs from inherently safer compounds, such as carboxylic acids, amino acids, and choline derivatives is essential [50,57,58,59,60,61,62,63,64,65] and their environmental footprint can be further reduced through effective reuse and recycling [66].

Currently, the application of ILs in microalgae lipid extraction, particularly from *Chlorella* species, has been investigated mostly for biodiesel production, with a predominant focus on commercially available imidazolium-based ILs [6,46,53,54,55,67,68,69,70,71,72,73,74,75,76,77,78]. Comparisons have been made with ILs derived from ammonium [34,35,44,79,80], phosphonium [35,81,82], pyridinium [35], pyrrolidinium [34,80], guanidinium, and DBU (1,8-diazabicyclo [5.4.0]undec-7-ene) [83] cations, while fewer studies have explored ILs exclusively from non-imidazolium cations [45,84,85,86,87,88,89,90], notably choline and its derivatives [87,88].

In the present work, lipid extraction was performed using a microwave-assisted ionic liquid (MAE–IL) approach. Microwave-assisted extraction (MAE) is an efficient technique to recover bioactive compounds from microalgae, as it uses microwave energy to heat the solvent in contact with biomass, ensuring uniform internal heating [2,34]. The rapid temperature rise and resulting pressure gradient disrupt hydrogen bonds and weaken or even rupture cell walls, facilitating solvent penetration and enhancing lipid release. Heating occurs via two mechanisms: dipole rotation and ionic conduction [91,92]. Thus, the efficiency of microwave absorption largely depends on solvent polarity, with strongly polar solvents showing greater absorption capacity [2]. Compared to conventional extraction methods, MAE provides higher extraction yields, fewer degradation products, greater efficiency, shorter extraction times, lower solvent consumption, and does not require prior sample drying [34,46,54]. Nevertheless, this technique also presents limitations, including relatively high energy demand, the need for post-extraction separation steps, and limited applicability for heat-sensitive compounds [92].

This study explores the use of IL and MAE, both individually and in combination, to enhance lipid extraction from *Chlorella vulgaris*. Their performance was compared with the traditional methods: Soxhlet, Folch, and Bligh & Dyer. The ionic liquid chosen features a choline-derived cation, part of the B-vitamin complex [58], providing biocompatibility and a hexanoic acid anion, a low-toxicity carboxylic acid. The recyclability of the IL was also addressed, as well as some critical parameters including solvent composition, biomass-to-solvent ratio, extraction time, microwave power, operating temperature, and water content. Combining MAE with ILs offers several advantages, including higher extraction yields, shorter extraction times, and reduced energy consumption [93].

## 2. Results and Discussion

### 2.1. Lipid Extraction Using Conventional Methods

**Figure 1:**
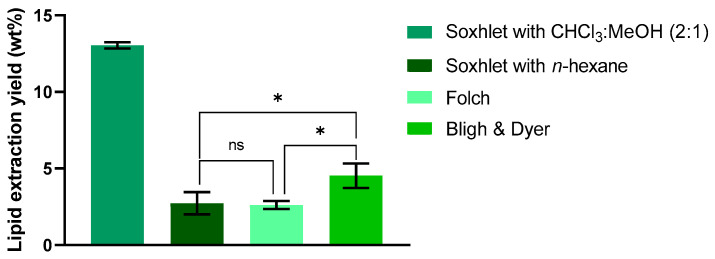
Lipid extraction yields obtained by different conventional methods, expressed as % of total algal biomass used. Data are expressed as the mean ± standard deviation of three independent experiments. Statistically significant differences are represented by asterisks: * *p* = 0.0190, ns—non-significant.

In order to isolate the total lipid fraction from *Chlorella vulgaris* and to rigorously assess the performance of the proposed optimized protocol, three well-established lipid extraction methodologies, Soxhlet, Folch, and Bligh & Dyer, were employed for comparative evaluation. As shown in Figure 1, lipid extraction yields varied considerably depending on the conventional method employed. The Soxhlet method with chloroform/methanol (2:1 *v*/*v*) achieved the highest yield (13.04%), approximately 4.8-fold greater than the obtained with Soxhlet using *n*-hexane.

This difference can be explained by solvent polarity. While hexane selectively extracts neutral lipids and minimizes co-extraction of polar compounds, resulting in a purer extract, polar solvents or mixtures such as chloroform/methanol can penetrate the polar phospholipid membrane (associated with proteins), breaking protein–lipid bonds and effectively extracting both neutral and polar lipids stored in lipid droplets. However, this broader solubilization also promotes the co-extraction of pigments and proteins, which may not be desirable in the final product [7,33,34,35]. Consequently, lipid yields obtained with chloroform/methanol (2:1) Soxhlet extraction may be overestimated. This is not only due to the presence of unwanted polar compounds but also to the fact that direct evaporation of the solvent mixture, without prior solvent separation (unlike the Folch and Bligh & Dyer protocols), leads to the retention of non-lipid polar compounds in the final extract.

Furthermore, both Folch and Bligh & Dyer methods produced lower lipid yields compared with Soxhlet extraction using chloroform/methanol. This discrepancy might arise from two main factors: (1) the use of an aqueous 0.88% NaCl solution to separate the chloroform and methanol phases, effectively removing polar impurities, and (2) the milder extraction conditions (room temperature, 10 min) relative to Soxhlet, which operates for 12 h at >70 °C.

### 2.2. Lipid Extraction from Biomass Residue (Sediment) with Chloroform/Methanol (1:1)

After evaluating the performance of the traditional methods for extracting lipids from algae, a more sustainable and improved procedure was developed using an ionic liquid with and without microwave-assisted extraction. The chemical and thermal characterization of the tested IL, [N_1 1 2OH 2OH_][C_6_H_11_O_2_], including ^1^H and ^13^C NMR, FTIR-ATR and DSC analyses, is presented in the Supplementary Materials (Figures S1–S4).

The synthesized IL [N_1 1 2OH 2OH_][C_6_H_11_O_2_] was selected due to its intermediate alkyl chain length, which provides a balanced polarity, avoiding the excessive polarity of shorter chains and the reduced polarity of longer ones, thus promoting lipid affinity while facilitating subsequent separation from the IL. Microwave irradiation was combined with this solvent to enhance cell wall disruption, thereby improving lipid extraction efficiency and enabling recovery of others bioactive compounds. For the extraction procedure, a mixture of chloroform and methanol (1:1, *v*/*v*) was used to obtain the results presented below, as detailed in Section 3.6.1. The overall extraction process is illustrated in the schematic provided in the Supplementary Materials (Figure S13).

**Figure 2:**
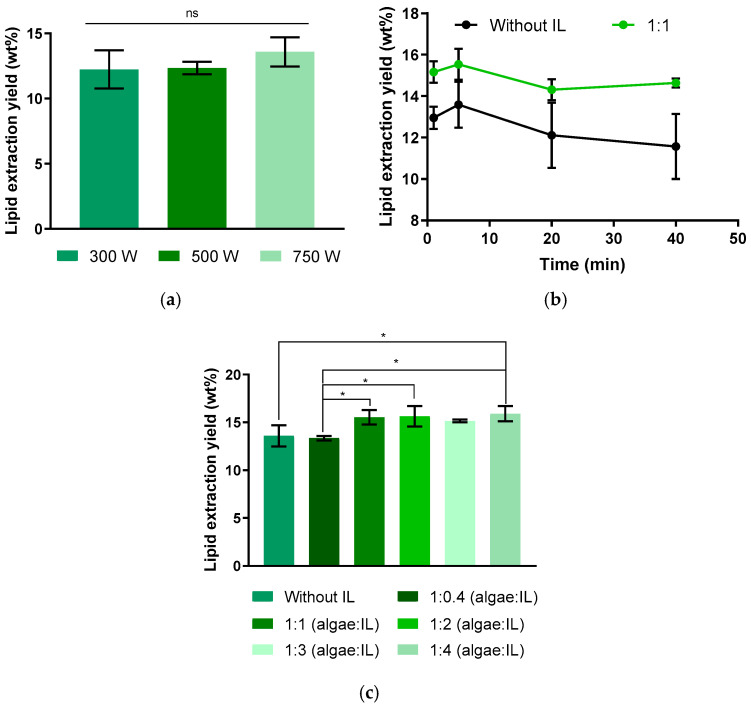
Influence of different microwave pretreatment variables on the lipid extraction yield from biomass sediment, using chloroform/methanol (1:1, *v*/*v*) as extraction solvent: (**a**) Microwave power (300, 500, and 750 W) on a 5 min treatment without IL. (**b**) Pretreatment time (1, 5, 20, and 40 min) with and without IL (1:1, *w*/*w*, algae/IL) at 750 W and 60 °C. (**c**) Algae/IL mass ratio during microwave pretreatment (without IL, 1:0.4, 1:1, 1:2, 1:3 and 1:4) at 750 W and 60 °C during 5 min. Yields are expressed as % of total algal biomass used. Data represent mean ± standard deviation of three independent experiments. Statistically significant differences are indicated by asterisks: * *p* = 0.0450. ns—non-significant.

Initially, the power parameter was optimized, with tests conducted without the incorporation of IL and a sample treatment time of 5 min in MW. As shown in Figure 2a, the results obtained revealed that there was no significant difference in the lipid yield when the power was varied. For the subsequent tests, 750 W was defined as the optimum power, which was also the approximate power used in other studies [34,46,54,68]. Furthermore, the ideal time for the sample to remain in the microwave was evaluated in the absence or in the presence of IL at a mass ratio of 1:1 (algae/IL). Figure 2b indicates that 5 min was the optimal extraction time. Finally, the mass ratio between *Chlorella vulgaris* and IL was investigated. The results obtained for the proportions 1:0.4, 1:1, 1:2, 1:3, and 1:4 (algae/IL) (*m*/*m*) are shown in Figure 2c. Although the yields show small but statistically significant differences as the algae/IL ratio increases, a modest rise in lipid yield is observed at the four highest IL concentrations.

### 2.3. Lipid Extraction from IL Phase (Supernatant)

The amount of lipids found in the supernatant obtained during biomass pretreatment was also quantified. This lipid fraction, extracted solely through the combined action of microwaves and the ionic liquid (IL), was considered as the lipid extraction during pretreatment.

Lipids were isolated from the supernatant using *n*-hexane, as illustrated in the schematic provided in Figure S14 (bottom) and further experimental details are provided in Section 3.7. The contact time between the two phases, during which vigorous stirring was applied, was evaluated. According to Figure S5, the optimal stirring time was determined to be six hours, and this duration was subsequently adopted to maintain phase contact under agitation.

**Figure 3:**
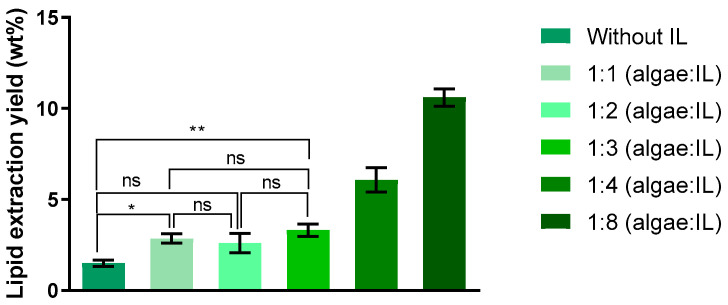
Influence of algae/IL mass ratio during microwave pretreatment (750 W, 5 min, 60 °C) on lipid content in the supernatant, expressed as % of the total algal biomass used. Data represent mean ± standard deviation of three independent experiments. Statistically significant differences are represented by asterisks: * *p* = 0.0499, ** *p* = 0.0054, ns—non-significant.

The results obtained clearly demonstrate the actual influence of the mass ratio (algae/IL) in the microwave pretreatment of *Chlorella vulgaris* on lipids extract obtained from supernatant (Figure 3). As there was an increase in the lipids present in the supernatant when going from a mass ratio of 1:3 to 1:4 (algae/IL), the biomass was also treated with 1:8 (algae/IL). When using mass ratios of up to 1:3 (algae/IL), the increase in lipid extraction yield is minimal or nearly negligible compared to the yield obtained without IL in pretreatment. However, when IL is added to the pretreatment at a mass ratio of 1:4 and 1:8 (algae/IL), the lipid content increases fourfold and sevenfold, respectively, compared to the pretreatment without IL. By using a 1:8 mass ratio (algae/IL), the highest lipid content is observed, reaching approximately 10.61 ± 0.39% (percentage of the total mass of algae used).

The results obtained reveal that a significant amount of lipids, or compounds with lipid affinity, is extracted from the algal biomass during the pretreatment. This suggests that these extracted components should be considered as part of an integrated process for the overall extraction of lipids from the algae.

To assess the impact of MW and IL, tests were performed with and without MW, as well as with and without IL, using algae/IL ratios of 1:1 and 1:4 (*m*/*m*). Figure S6 provides a direct comparison of lipid content with and without microwave radiation, both in the absence or in the presence of IL at two different mass ratios. The results indicate that, as the algae/IL ratio increases, the impact of microwave radiation on lipid yield becomes significantly more pronounced. When a mass ratio of 1:4 (algae/IL) is used, microwave pretreatment leads to an approximately 1.7-fold increase in lipid extraction yield compared to pretreatment without microwave application.

**Figure 4:**
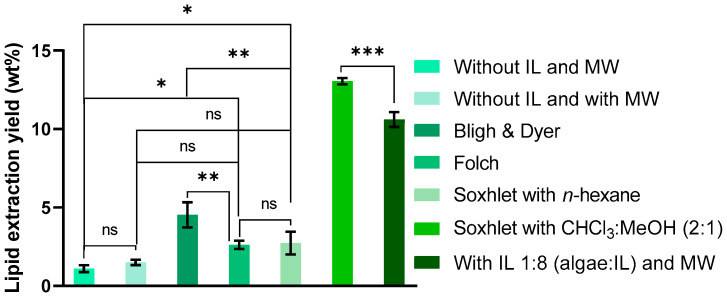
Comparison of lipid extraction yield from the supernatant under different conditions: without pretreatment (absence of IL and MW), with MW pretreatment without IL, with MW pretreatment and IL at a 1:8 mass ratio (algae/IL) and using four conventional extraction methods. Yields are expressed as % of total algal biomass used. Data represent mean ± standard deviation of three independent experiments. Statistically significant differences are represented by asterisks: * *p* = 0.0204, ** *p* = 0.0053, *** *p* = 0.0003, ns—non-significant.

Figure 4 illustrates a comparison between the results obtained using the 1:8 mass ratio (algae/IL), which provided the highest lipid extraction yield, and those from various conventional extraction methods and control conditions, including pretreatment without microwaves and IL, as well as with microwaves and without IL.

Compared to the Bligh & Dyer method, which is the most widely used in the literature for total lipid extraction and method comparison, the 1:8 (algae/IL) ratio resulted in an increase in lipid yield from 4.53 ± 0.65% to 10.61 ± 0.39%.

It is important to note that the method tested here accounts solely for lipids present in the supernatant itself, without direct solvent extraction (e.g., hexane or chloroform/methanol) from the algae, as performed in the conventional methods. This suggests that the 1:8 (algae/IL) ratio could potentially extract even more lipids, since pretreatment facilitates cell wall disruption and IL penetration. However, some lipids may remain within the microalgae and not fully transfer into the IL during pretreatment. Results for the extraction of residual lipids still present in the pretreated algae will be presented in the following section. The tested IL, composed of a polar cation and a significantly less polar anion, enables the extraction of both polar and non-polar lipids, like mixtures of polar and non-polar solvents [7,33,34,35].

#### Characterization of the Extract by ^1^H NMR

**Figure 5:**
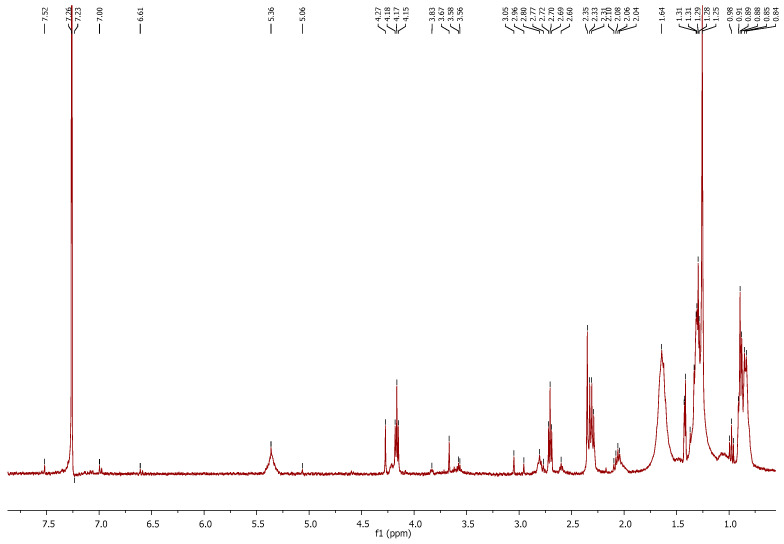
^1^H NMR of the extract obtained using [N_1 1 2OH 2OH_][C_6_H_11_O_2_] in the mass ratio 1:8 (algae/IL).

The analyzed extract, being the crude extract obtained directly from the extraction process, consists of a complex mixture of various lipid compounds, including triglycerides, diglycerides, monoglycerides, and free fatty acids. Figure 5 shows the spectrum obtained, which is in accordance with the NMR reported for other complex lipid mixtures [94]. Briefly, the chemical shift between 0.81 ppm and 0.86 ppm corresponds to the protons of the methyl group (C**H_3_**) in saturated and monounsaturated ω-9 and/or ω-7 acyl groups and fatty acids (FA). The peaks between 0.88 ppm and 0.91 ppm also correspond to the C**H_3_** group but are associated with unsaturated ω-6 acyl groups and FA, while unsaturated ω-3 acyl groups and FA appear at a chemical shift of 0.96–1.00 ppm. The chemical shift between 1.25 ppm and 1.43 ppm corresponds to the -(C**H_2_**)_n_- protons of acyl groups and FA. At a chemical shift between 1.59 ppm and 1.69 ppm, the protons (-C**H_2_**CH_2_COOH or -C**H_2_**CH_2_OCO-) corresponding to the acyl groups in triglycerides, diglycerides, monoglycerides, and fatty acids can be observed. Around 2.06 ppm, the spectrum reveals the presence of -C**H_2_**CH=CH- protons associated with acyl groups and fatty acids. Between 2.28 ppm and 2.35 ppm, additional protons (-CH_2_C**H_2_**COOH, -CH_2_C**H_2_**OCO-) linked to the acyl groups in triglycerides, diglycerides, monoglycerides, and fatty acids are detected, further confirming the composition of the lipid extract. The chemical shift at 2.70 ppm (well-defined triplet) corresponds to the protons (=HC-C**H_2_**-CH=) of diunsaturated ω-6 acyl groups and fatty acids. Meanwhile, the peaks observed between 2.77 ppm and 2.84 ppm represent the protons (=HC-C**H_2_**-CH=) of polyunsaturated ω-6 and ω-3 acyl groups and fatty acids, respectively. The peaks corresponding to chemical shifts between 3.56 ppm and 3.84 ppm are associated with the protons (ROCH_2_-CHOH-C**H_2_**OH; ROCH_2_-CH(OR′)-C**H_2_**OH; HOC**H_2_**-CH(OR)-C**H_2_**OH; ROCH_2_-C**H**OH-CH_2_OH) of the glycerol group in 1-monoglyceride, 2-monoglyceride, and 1,2-diglyceride. Peaks around 4.15 ppm and 4.27 ppm correspond to protons (ROC**H_2_**-C**H**OH-C**H_2_**OR′; ROC**H_2_**-CHOH-CH_2_OH; ROC**H_2_**-CH(OR′)-C**H_2_**OR″; ROC**H_2_**-CH(OR′)-CH_2_OH) from the glycerol group in 1-monoglyceride, 1,2-diglyceride, 1,3-diglyceride, and triglycerides. The barely visible peaks around a chemical shift of 5.06 ppm correspond to protons (HOCH_2_-C**H**(OR)-CH_2_OH; ROCH_2_-C**H**(OR′)-CH_2_OH; ROCH_2_-C**H**(OR′)-CH_2_OR″) of the glycerol group in 2-monoglyceride, 1,2-diglyceride, and triglycerides. Finally, the chemical shift around 5.36 ppm is associated with protons -C**H**=C**H**- of the double bonds in acyl groups and free fatty acids. Due to the complexity of the sample, a more exhaustive NMR analysis would be required to accurately identify the fatty acids and determine their specific forms.

### 2.4. Total Lipid Extraction (Supernatant and Biomass Sediment)

With the objective of extracting lipids from the biomass after pretreatment with MW and IL, the conventional chloroform/methanol system previously tested was substituted with a more environmentally benign solvent mixture, ethyl acetate/ethanol (1:1 *v*/*v*). This choice was motivated by the fact that both solvents are considered suitable for use, according to solvent selection guides, based on safety, health, and environmental criteria aligned with the Globally Harmonized System and European regulations, whereas chloroform is classified as highly hazardous [95]. The overall extraction process is illustrated in the schematic provided in Figure S14 and further experimental details are provided in Section 3.6.2.

**Figure 6:**
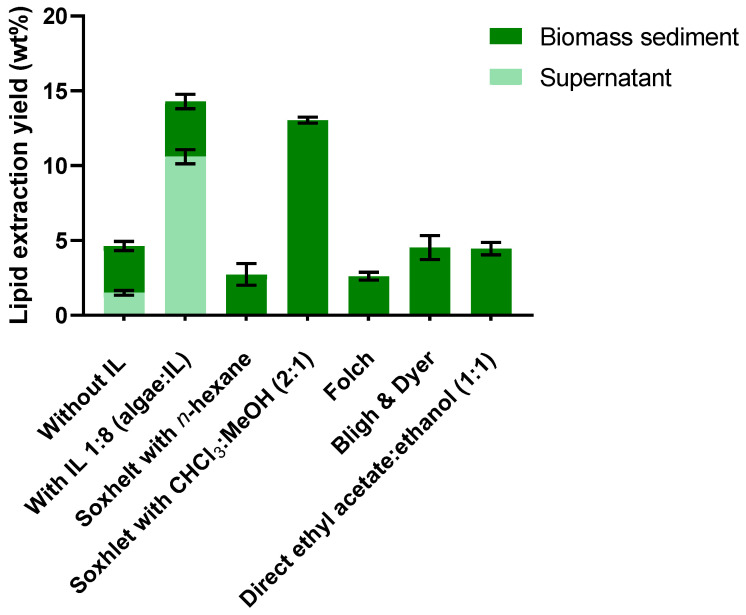
Total lipid extraction yield, including lipids recovered from both supernatant and biomass sediment, obtained with and without IL treatment (with MW), conventional methods, and direct extraction using ethyl acetate/ethanol (1:1). Yields are expressed as % of total algal biomass used. Data represent mean ± standard deviation of the three independent experiments.

Figure 6 presents the total lipid extraction yield for *C. vulgaris*, including both the lipids recovered from the supernatant and those extracted from the pretreated biomass. Direct ethyl acetate/ethanol (1:1) represents the lipid yield obtained through direct extraction from the microalgae using an ethyl acetate/ethanol (1:1 *v*/*v*) solvent mixture, without any prior pretreatment. For comparative analysis, the results obtained using four conventional extraction methods tested in this work for *C. vulgaris* are also presented in Figure 5.

Analyzing the results in Figure 6, the lipid yield extracted from biomass pretreated (extraction in the biomass sediment) without IL was 3.12 ± 0.25%, a value like that obtained from extraction in the supernatant. In the case of algae pretreated with IL, 3.69 ± 0.40% of lipid compounds were extracted. The total lipid yield (from both the supernatant and sedimented biomass) obtained with IL treatment was 14.29 ± 0.75%, more than twice that achieved with MW treatment alone. It was also slightly higher than the yield obtained using the Soxhlet method with CHCl_3_:MeOH (2:1) (13.04 ± 0.16%), and significantly superior to the yields from all other methods tested.

### 2.5. Recovery of Ionic Liquid

On average, 84.80 ± 7.18% of the IL employed in the extraction was successfully recovered across. When reused, IL [N_1 1 2OH 2OH_][C_6_H_11_O_2_] exhibited no significant difference in lipid extraction yield compared with pure IL (11.22 ± 0.73% vs. 10.61 ± 0.39%).

**Figure 7:**
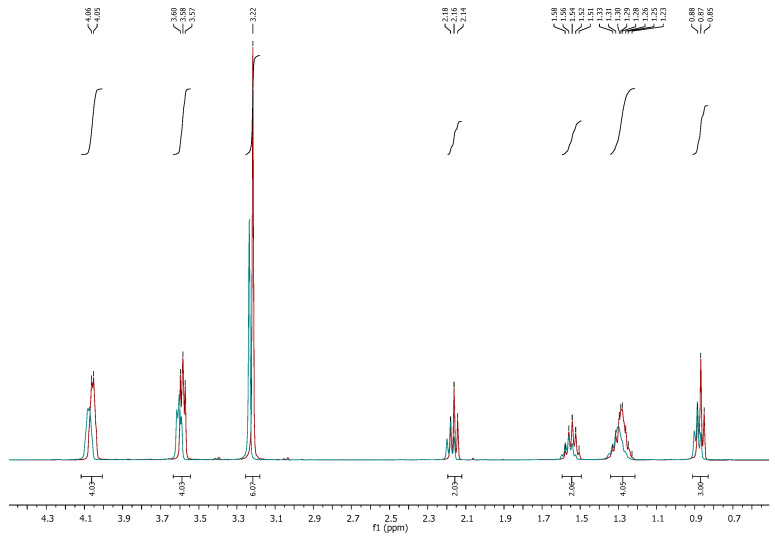
^1^H NMR of pure IL [N_1 1 2OH 2OH_][C_6_H_11_O_2_] (blue) and reused IL (red) from extraction with a 1:8 mass ratio (algae/IL).

Characterization of the recovered IL by ^1^H NMR, DSC, and FTIR confirmed that it maintained its structural integrity despite a slight coloration, likely attributable to residual pigments such as chlorophylls [35]. As shown in Figure 7, the ^1^H NMR spectrum of the reused IL (red) overlapped with that of the pure IL (blue), demonstrating the absence of relevant contamination and confirming its suitability for reuse.

The glass transition temperature of the recovered IL [N_1 1 2OH 2OH_][C_6_H_11_O_2_] (−78.52 °C) was nearly identical to that of the pure IL (−80.07 °C), confirming the preservation of its physicochemical properties (Figure S7). Similarly, the FTIR-ATR spectra (Figure S8) demonstrated that IL recovered from extractions performed at different mass ratios (1:1, 1:4, and 1:8, algae/IL) displayed the same characteristic vibrational bands as the pure IL, further validating its structural integrity.

**Figure 8:**
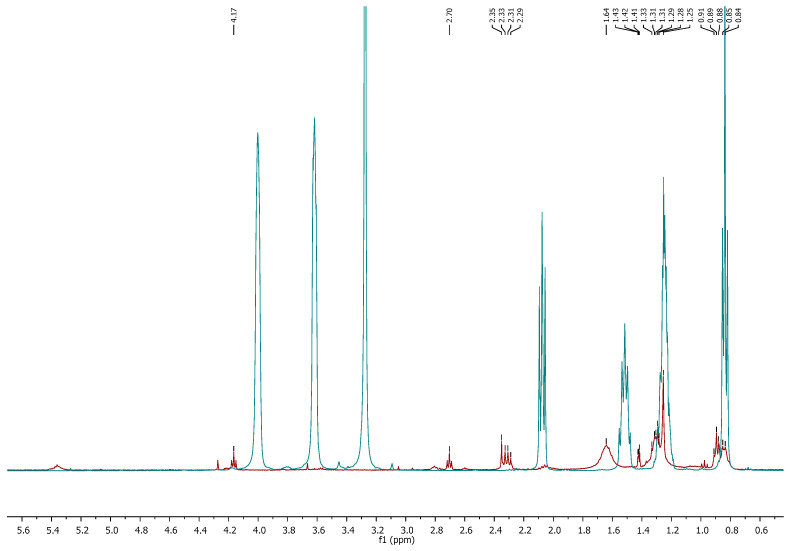
^1^H NMR of the extract obtained using [N_1 1 2OH 2OH_][C_6_H_11_O_2_] in the mass ratio 1:8 (algae/IL) (red) and [N_1 1 2OH 2OH_][C_6_H_11_O_2_] pure (blue).

To confirm the effective separation of lipids from the IL, the obtained extracts, from the supernatant, were analyzed by ^1^H NMR and FTIR-ATR. As illustrated in Figure 8, the ^1^H NMR spectrum of the lipid extract obtained after MW-assisted pretreatment with IL at a mass ratio of 1:8 (algae/IL) showed no detectable IL signals. This result, further supported by FTIR analysis (Figure S9), confirms the absence of [N_1 1 2OH 2OH_][C_6_H_11_O_2_] in the lipid extract, demonstrating the effective separation of lipids from the IL.

FTIR analysis of the extracts obtained at algae/IL ratios of 1:4 and 1:8 (Figure S9) revealed no evidence of residual [N_1 1 2OH 2OH_][C_6_H_11_O_2_]. Although some spectral similarities were expected due to the presence of hexanoic acid in the IL, the results confirm that the extracts were free of IL contamination. The weak peaks around 3000 cm^−1^ and 1658 cm^−1^ correspond to C=C stretching vibrations, while the broad OH band near 3200 cm^−1^, prominent in the IL spectrum, was markedly reduced in the extracts, reflecting the absence of the hygroscopic IL. In contrast, the stronger band at ~1740 cm^−1^, attributed to C=O stretching, further distinguished the lipid extracts from the IL [96,97,98,99,100,101].

### 2.6. Fatty Acid Methyl Esters (FAMEs) Composition Analysis

The results of the FAME composition analysis for the species tested, as well as for the different methods and conditions evaluated, are presented below.

In the following figures, “Others” refers to a group that includes the following methyl esters of the compounds: butyric acid (C4:0), caproic acid (C6:0), capric acid (C10:0), lauric acid (C12:0), myristic acid (C14:0), pentadecanoic acid (C15:0), 15-Methylpalmitic acid, a mix of compounds to C17-derived FAMEs, Cascarillic acid, 8,11,14-heptadecatrienoic acid, (C17:3 cis) and margaric acid (C17:0).

#### 2.6.1. FAMEs Composition Obtained from IL Phase (Supernatant)

**Figure 9:**
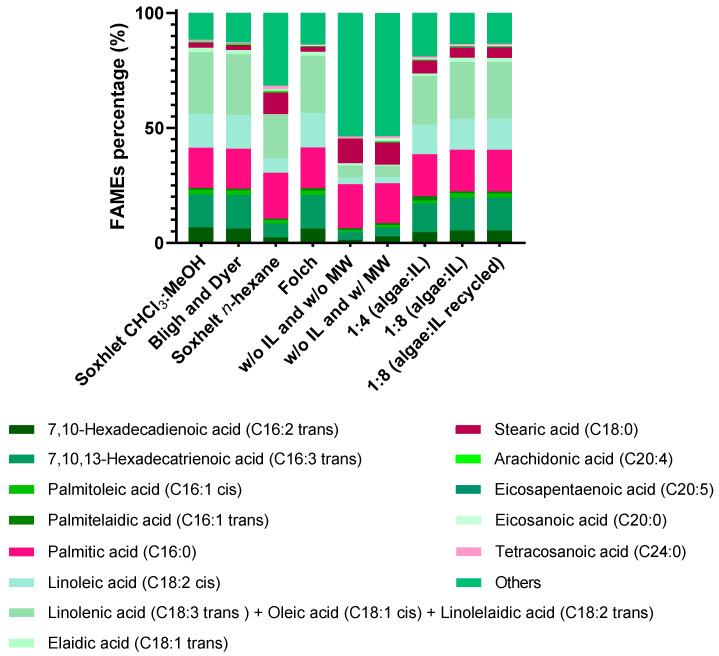
FAMEs profile of *C. vulgaris* (supernatant), obtained using conventional methods and under different studied conditions. Data are expressed as the mean ± standard deviation of three independent experiments.

Figure 9 illustrates that increasing the algae-to-IL mass ratio results in a FAME profile (relative percentage of each FAME) that more closely resembles that obtained through conventional methods, particularly at a 1:8 (algae/IL) ratio. Additionally, when recycled IL is used, the FAME profile remains largely unchanged compared to pure IL.

At a 1:8 mass ratio (algae/IL) combined with MW for just 5 min as a pretreatment, approximately 57% of the FAMEs obtained by the Soxhlet method with chloroform/methanol (2:1) (43.10 mg/g; Table 1 and Figure S10) were extracted from the supernatant phase, corresponding to 24.46 mg/g (Table 2 and Figure S10). In comparison, the Bligh & Dyer method, traditionally used as a benchmark, yielded 17.41 mg/g (Table 1 and Figure S10). The use of recycled IL at the same 1:8 mass ratio slightly increased the FAME yield to 25.08 mg/g (Table 2 and Figure S10), indicating that recycling the IL does not significantly affect extraction efficiency.

The amounts of each FAME obtained by conventional extraction methods and those recovered from the supernatant of *C. vulgaris* using different conditions are reported in Table 1 and Table 2, respectively.

#### 2.6.2. FAMEs Total Composition Obtained (Supernatant and Biomass Sediment)

**Figure 10:**
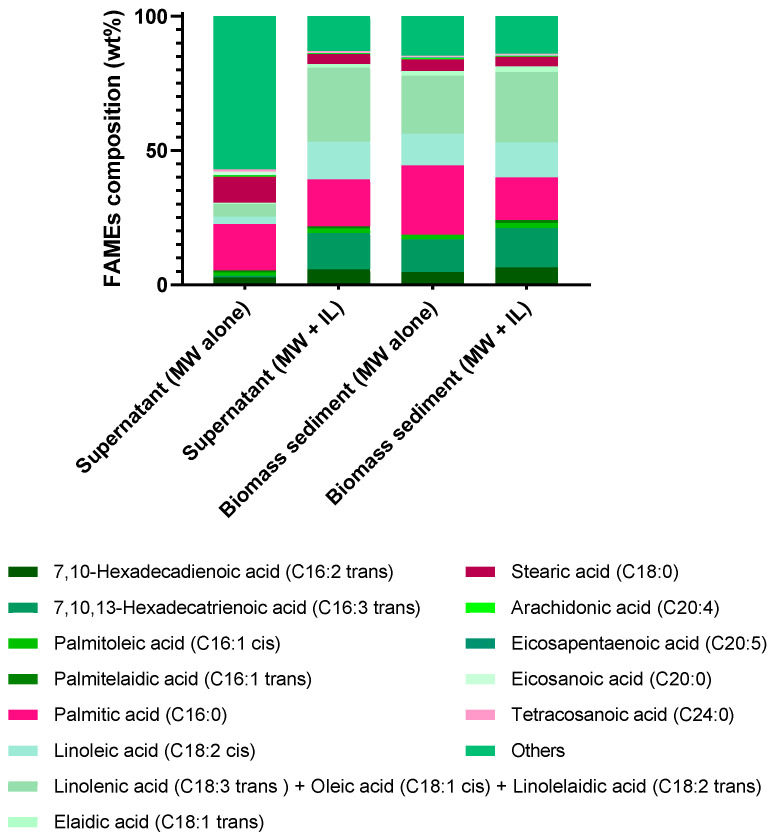
Comparison of the FAMEs profile obtained from the supernatant and biomass sediment in the MW treatment of *C. vulgaris*, with and without IL. Data are presented as the mean ± standard deviation of three independent experiments.

Figure 10 and Figure S11 present the FAME profiles and yields, respectively, obtained from the supernatant and biomass sediment of *C. vulgaris* after MW pretreatment in the presence or absence of [N_1 1 2OH 2OH_][C_6_H_11_O_2_] at a 1:8 mass ratio (algae/IL). The FAME profile in the supernatant obtained without IL differed substantially from that obtained with IL and from the profiles in the sedimented biomass. By contrast, the FAME profiles of the biomass sediment showed high similarity between treatments with and without IL. Importantly, the use of IL resulted in consistent FAME profiles in both supernatant and biomass fractions, indicating that IL does not alter lipid composition but enhances extraction efficiency. Quantitatively, 18.09 ± 0.35 mg FAMEs/g algae were recovered from the biomass sediment after MW pretreatment with IL, compared with 16.32 ± 0.45 mg FAMEs/g algae without IL (Figure S11 and Table 3).

A distinct FAME profile observed in the supernatant obtained without IL (first bar in Figure 10) can be attributed to differences in compounds affinity during the pretreatment. In the absence of IL, only water interacts with the algae, favoring the extraction of more hydrophilic compounds, which are predominantly shorter-chain FAMEs categorized as “others” in the figure. In contrast, when IL is used, it enables the extraction of less polar compounds with higher affinity for the IL, such as longer-chain FAMEs (C18 and above). Consequently, in the biomass fraction after water-only pretreatment, most short-chain compounds have already been removed, resulting in a FAME profile that is more like that obtained with IL-assisted treatment (third and fourth bar in Figure 10).

The total FAME yield obtained from both phases (supernatant and biomass sediment) after MW pretreatment combined with [N_1 1 2OH 2OH_][C_6_H_11_O_2_] reached 42.56 ± 0.64 mg/g algae. This value is comparable to that achieved with the conventional Soxhlet method using chloroform/methanol (2:1) (43.10 ± 0.21 mg/g algae) and exceeds the yields obtained with the Bligh & Dyer method, direct extraction with ethyl acetate/ethanol (1:1), and MW pretreatment alone by 2.4-, 2.7- and 2.0-fold, respectively (Figure S12). The detailed distribution of individual FAMEs extracted from the biomass sediment of *C. vulgaris* is provided in Table 3. 

Based on the results of this study, it can be concluded that microwave irradiation and ionic liquids act synergistically to improve lipid accessibility through the simultaneous acceleration of cell-wall deconstruction and mass transfer. Microwave pretreatment is one of the most effective methods for disrupting microalgal cells prior to lipid extraction. Microwaves interact directly with polar molecules, such as water, converting electromagnetic energy into heat through dipole rotation. This leads to rapid and uniform volumetric heating within the biomass, generating localized pressure and temperature gradients that rupture the cell walls and enhance lipid release. The process increases solvent penetration, reduces extraction time, and minimizes solvent use while avoiding significant thermal degradation of lipids [102].

Beyond these thermal and physical effects, the presence of ionic liquids further enhances the disruption process through chemical interactions. ILs can disrupt the hydrogen-bonded polysaccharide network of the microalgal wall via strong Coulombic and hydrogen-bonding forces. These interactions can, in some systems, promote partial hydrolysis or dissolution of wall components without added catalysts at 100–140 °C, thereby facilitating the release of intracellular contents [73].

In the work by S Rezaei Motlagh et al. [103], IL-microwave-assisted extraction studies on *Nannochloropsis gaditana* evidenced cleavage of glycosidic linkages and marked surface disruption of the cell walls, consistent with increased IL penetration and lipid diffusion. The same work reported multi-fold gains in total lipid and EPA yields versus Soxhlet, aligning with the above physicochemical picture.

As previously mentioned, the lipid and FAME content in *Chlorella vulgaris* is highly influenced by cultivation conditions, which makes direct comparisons with results from the literature challenging. However, the results obtained in this study were significantly higher than those reported by Kim et al. [74], in which biomass pretreated with [Bmim][MeSO_4_] combined with ultrasound yielded only 74 mg/g dry cell weight of lipids. In contrast, under optimal conditions in the present study, it was possible to extract approximately 143 mg/g. The FAME content was also superior, reaching 42.56 mg/g (total), compared to 33.3 mg/g dry cell weight reported in the referenced study. In the study by Olkiewicz et al. [85], the lipid yield obtained using [P(CH_2_OH)_4_]Cl treatment (8.1%) was lower than the yields achieved in the present study (14.29%). Additionally, the FAME content reported in their study was comparable to that obtained in this work. In the study by Krishnan et al. [68], the FAME yields obtained using three different ILs ([Omim]Br, [Omim][OAc] and [Omim][NTf_2_]) in combination with MW radiation were markedly lower than those achieved in the present study. Moreover, the lipid contents achieved by those authors (~13.5, 19.5 and 8.5% for the respective ILs) were also inferior to the total lipid recovery observed here, considering both supernatant and biomass sediment fractions (14.29%). Kim et al. [55] reported that ILs [Bmim][CF_3_SO_3_], [Emim][MeSO_4_], [Bmim][MeSO_4_], and [Emim]Cl exhibited higher selectivity for FAME extraction, ranging from 73.09 to 111.36 (mg/g DW), as evidenced by greater FAME yields compared to the present work. However, total lipid recovery was lower than that obtained here. Furthermore, ILs with imidazolium cations are generally more toxic and less biodegradable than ILs with choline-derived cations [50,57,58,59,60,61,62,63,64,65]. In the work of Krishnan et al. [71], some of [Omim]Br (~24% lipids recovery at an IL concentration of 2.5%) and [Omim][OAc] (~22% lipids recovery at 2.5%) tested led to higher lipid extraction efficiency than observed in this study. Choi et al. [6] demonstrated that treatments based on other imidazolium salts resulted in superior lipid (~40–250 mg/g cell) and FAME (~940 mg/g lipid) recoveries compared to those achieved in this study. Nevertheless, it is noteworthy that their process was performed at higher temperatures (120 °C) and for longer time (2 h), which may account for the enhanced extraction performance.

Table S1 summarizes the mass balance for the lipids and FAMEs obtained in this study, allowing a direct comparison between the traditional methods and MAE-IL approach. Overall, using the ionic liquid as a pretreatment enabled the valorization of the supernatant, a fraction rarely explored in the literature but notably rich in lipids (10.61 ± 0.39% of the total 14.29 ± 0.75% obtained at a 1:8 (m/m) ratio, 5 min, 750 W, 60 °C) and in FAMEs (24.46 ± 0.48 mg/g algae). This approach clearly outperformed the conventional methods, yielding higher lipid extraction (14.29% vs. 2.61–4.53%). Although Soxhlet extraction with chloroform and methanol provided the best lipid yield (13.04 ± 0.16%) in a single step, the overall procedure requires the use of hazardous solvents whose use is increasingly discouraged.

## 3. Materials and Methods

### 3.1. Materials

*Chlorella vulgaris* was supplied by ALLMICROALGAE—Natural Products, S.A. (Pataias, Portugal). The microalgae used in this study were cultivated under sustainable autotrophic conditions, relying on sunlight and carbon dioxide (CO_2_) as energy and carbon sources, respectively, while releasing oxygen as a byproduct. Diethyl ether (≥99.8%), chloroform (99.0–99.4%), methanol (≥99.8%), and sodium sulfate (≥99.0%) were purchased from Honeywell—Riedel-de-Haën (Seelze, Germany). 2-chloroethanol (99.0%), tetradecane (≥99.0%), nonane (≥99.8%), boron trifluoride–methanol solution (14% in methanol) and Supelco 37 Component FAME Mix standard (CRM47885) were acquired from Sigma-Aldrich (Darmstadt, Germany). Hexanoic acid (>98%) and 2-dimethylaminoethanol (≥99.0%) were achieved from TCI chemicals (Zwijndrecht, Belgium). Acetone (for analysis), acetonitrile (for analysis), and *n*-hexane (≥99.0%) were purchased from Carlo Erba Reagents (Val-de-Reuil, France), deuterium oxide (99.9%), dimethylsulfoxide D_6_ (99.8%), and chloroform D (99.8%) were achieved from Eurisotop (Saint-Aubin, France). Amberlite^TM^ IRN-78, *n*-hexane, sodium hydroxide, and sodium chloride (for analysis) were obtained from Alfa Aesar (Ward Hill, MA, USA), Valente e Ribeiro Lda. (Alcanena, Portugal), LabChem (Zelienople, PA, USA), and PanReac Applichem (Barcelona, Spain), respectively.

### 3.2. Synthesis of Ionic Liquid

#### 3.2.1. Synthesis of [N_1 1 2OH 2OH_]Cl

2-Dimethylaminoethanol was mixed with 1.1 equiv. of alkylating agent, 2-chloroethanol, and *n*-hexane in a 100 mL pressure reaction vessel. This mixture was kept at 80–90 °C for 24 h. The ionic liquid was thoroughly washed with diethyl ether to remove the unreacted alkylating agent. Ionic liquid was then dried in the vacuum for 1–2 days, and its purity confirmed by ^1^H and ^13^C NMR. This IL was obtained in 95–99% yield. Chemical structure of [N_1 1 2OH 2OH_][C_6_H_11_O_2_] is shown in Figure S15.

#### 3.2.2. Synthesis of [N_1 1 2OH 2OH_][C_6_H_11_O_2_] via Metathesis Reaction

An aqueous solution of *N*-alkyl derivative cholinium chloride was slowly passed through an anion exchange column Amberlite^TM^ IRN-78 and the corresponding hydroxide solution was added and stirred at room temperature to a solution of hexanoic acid (1.0 equiv.) diluted in a mixture of water and acetone. The water and acetone were then removed from the reaction mixture by evaporation. Ionic liquid was washed with diethyl ether and dried in high vacuum for 2 days at 40–50 °C to guarantee minimum water content. Moreover, AgNO_3_ test was used to confirm the absence of halogen presence in the final IL. IL was characterized by ^1^H and ^13^C NMR, FTIR-ATR, and DSC (Figures S1–S4).

### 3.3. Analytical Techniques

#### 3.3.1. Nuclear Magnetic Resonance (NMR)

^1^H and ^13^C NMR spectra were recorded on a Bruker Avance III 400 MHz spectrometer (Bruker, Bremen, Germany) using DMSO-*d*_6_, CDCl_3_-*d*, or D_2_O as a deuterated solvent. Chemical shifts (*δ*) are given in ppm and coupling constants (*J*) are given in Hz. The multiplicities of signals in ^1^H NMR are given with chemical shifts (s = singlet, t = triplet, qt = quartet, and m = multiplet).

#### 3.3.2. Attenuated Total Reflectance-Fourier Transform Infrared (FTIR-ATR)

Fourier transform infrared (FTIR) spectra over the range 400–4000 cm^−1^ were collected at room temperature using a Cary 630 FTIR spectrometer equipped with a diamond attenuated total reflectance (ATR) of Agilent Technologies (Santa Clara, CA, USA), with a thermoelectrically cooled dTGS detector and KBr standard beam splitter. All the spectra were recorded via ATR method with a resolution of 1 cm^−1^, 16 scans.

#### 3.3.3. Differential Scanning Calorimetry (DSC)

The calorimetric experiments were carried out with a DSC Q2000 from TA Instruments Inc., New Castle, DE, USA (Tzero DSC technology) operating in the Heat Flow T4P option. Measurements were performed under anhydrous high purity nitrogen at a flow rate of 50 mL/min. The DSC Tzero calibration was carried out in the temperature range from −90 to 200 °C. Roughly, 10 mg of IL was encapsulated in TA Tzero a hermetic aluminum pan with a lid. The procedure consisted of 3 consecutive cooling and heating cycles: (a) cooling ramp to −90 °C at 10 °C/min; (b) heating ramp at 10 °C/min to a temperature of 160 °C. Glass transition temperature (T_g_) was determined on the second heating run.

### 3.4. Lipid Extraction Using Conventional Methods

To extract the total lipids from the studied *Chlorella vulgaris* and to evaluate the efficiency of the optimized method proposed, three conventional lipid extraction methods were employed: Soxhlet, Folch, and Bligh & Dyer.

#### 3.4.1. Soxhlet Extraction Method

Soxhlet apparatus was used for the oil extraction from microalgae using hexane or chloroform/methanol 2:1 (*v*/*v*) as solvent [36]. Oil was extracted from 0.5 g of dried biomass placed in a cellulose thimble using 50 mL of either hexane or chloroform/methanol (2:1, *v*/*v*) as solvent. The system was refluxed for 12 h (~70 cycles). After extraction, the solvent was evaporated at 40 °C using a rotary evaporator (BUCHI Rotavapor R-300, BUCHI, New Castle, DE, USA), and the residue was dried under vacuum until constant weight for gravimetric lipid quantification.

#### 3.4.2. Folch Extraction Method

Lipid extraction was performed following the Folch method [37,104]. Briefly, 0.5 g of dried biomass was vortexed with chloroform/methanol (2:1 *v*/*v*; 20:1 solvent-to-sample ratio) for 10 min. The mixture was filtered and rinsed twice with the same solvent. The combined filtrate was transferred to a separatory funnel and a final chloroform/methanol/water ratio of 8:4:3 was achieved by adding 0.88% NaCl solution. The mixture was stirred for 10 min and allowed to separate into two phases. The lipid-rich chloroform phase (lower layer) was collected and concentrated by rotary evaporation at 40 °C. The extract was transferred to a pre-weighed flask and dried under vacuum at 40 °C to constant weight. Lipid content was determined gravimetrically.

#### 3.4.3. Bligh & Dyer Extraction Method

Bligh & Dyer extraction was performed following the original procedure [38]. Briefly, 0.5 g of dry biomass was mixed with chloroform, methanol, and Milli-Q water at a ratio of 1:2:0.8 (*v*/*v*/*v*) and homogenized for 5 min. Additional chloroform was then added, and the mixture was homogenized for another 5 min. The mixture was filtered, and the biomass was re-extracted twice under the same conditions. The combined extracts were transferred to a separatory funnel and 0.88% NaCl solution was added to achieve final solvent proportions of 2:2:1.8 (chloroform/methanol/water, *v*/*v*/*v*). After phase separation, the lipid-rich chloroform phase (lower layer) was collected and concentrated by rotary evaporation at 40 °C. The extract was transferred to a pre-weighed flask and dried under vacuum at 40 °C to constant weight. Lipid content was determined gravimetrically.

### 3.5. Pretreatment of Chlorella vulgaris with IL and Microwave-Assisted Extraction

A microwave digestion system (Milestone Ethos 1600 Microwave Labstation, Milestone, Sorisole, Italy) equipped with a six-vessel carousel was used for the experiments. One vessel was fitted with a built-in temperature probe to monitor and control the reaction temperature throughout the process. Each poly(tetrafluoroethylene) (PTFE) vessel was provided with a single-port lid equipped with a pressure relief valve to regulate any excessive pressure. The system operated at a frequency of 2.45 GHz, and different microwave power levels (300, 500, and 750 W) were tested to optimize the operating conditions, together with four irradiation times (1, 5, 20, and 40 min), all performed at 60 °C. Power optimization was conducted without IL, whereas time optimization was performed both without IL and with IL at a 1:1 mass ratio (algae/IL). Subsequently, the algae-to-IL mass ratio was evaluated at 1:0.4, 1:1, 1:2, 1:3, 1:4, and 1:8. Each assay contained 0.5 g of *C. vulgaris* mixed with 0.4 mL (80% of algal biomass weight) of water or an aqueous solution of [N_1 1 2OH 2OH_][C_6_H_11_O_2_] at the desired proportion.

### 3.6. Lipid Extraction from Biomass Residue (Sediment)

After cooling, the suspension was centrifuged (VWR Mega Star 600R, Thermo Fisher Scientific, Waltham, MA, USA) at 9500 rpm for 20 min, and the supernatant was collected. The biomass pellet was then washed with 10 mL of Milli-Q water under agitation for 1 h, followed by a second centrifugation under the same conditions. The resulting supernatant was combined with the first, and the procedure was repeated twice more to ensure complete removal of residual ILs.

#### 3.6.1. Lipid Extraction from Biomass Residue (Sediment) Using Chloroform/Methanol (1:1 *v*/*v*) Mixture

Subsequently, 10 mL of chloroform/methanol (1:1 *v*/*v*) was added to the pretreated biomass sediment and stirred at room temperature for 1 h. The mixture was centrifuged, and the interphase was collected. This procedure was repeated twice, and the three supernatants were combined. The solvent was removed by rotary evaporation at 40 °C, and the resulting lipid extract was transferred to pre-weighed vials and dried under vacuum at 40 °C to constant weight. Lipid content was determined gravimetrically using Equation (1). The extraction process is illustrated in Figure S13.

(1) $$percentage\;lipid\;extract=\frac{m_{lipid\;extract}}{m_{biomass}}\times 100\%$$

#### 3.6.2. Lipid Extraction from Biomass Residue (Sediment) Using Ethyl Acetate/Ethanol (1:1 *v*/*v*) Mixture

To replace the conventional chloroform/methanol (1:1 *v*/*v*) system, a greener solvent mixture of ethyl acetate/ethanol (1:1 *v*/*v*) was employed, since both solvents are recommended for use, whereas chloroform is classified as highly hazardous [95]. Pretreated microalgae were vacuum-dried at 30 °C for 1 h and extracted with ethyl acetate/ethanol (1:40 *m*/*v*) under stirring at 50 °C for 2 h. After centrifugation, the supernatant was collected, and the biomass was re-extracted twice under the same conditions. The combined extracts were mixed with twice the volume of an aqueous NaCl solution (0.88% *w*/*v*), and additional ethyl acetate was added to achieve a final water-to-ethyl acetate ratio of 1:2 (*v*/*v*). The ethyl acetate phase, enriched in lipids, was recovered, concentrated by rotary evaporation at 40 °C, and dried under vacuum to constant weight in pre-weighed flasks. The process is represented in Figure S14 (top).

### 3.7. Lipid Extraction from IL Phase (Supernatant)

To quantify the lipids released during the pretreatment step, the supernatant (including the aqueous solutions from biomass washing) was extracted with *n*-hexane. Hexane was selected due to its high solubilization capacity for lipids and immiscibility with the ionic liquid (IL) used, thereby ensuring selective lipid recovery without IL contamination. The extraction time was optimized by evaluating stirring durations of 3, 6, 24, and 48 h. Under optimized conditions, an equivalent volume of *n*-hexane (~60 mL) was added to the collected supernatant and vigorously stirred. Every 2 h, the organic (upper) phase was separated in a separating funnel, and fresh hexane was added to the aqueous phase. All collected organic phases were pooled and concentrated by rotary evaporation at 40 °C. The lipid residue was transferred to pre-weighed vials and dried under vacuum (40 °C) to constant weight. Lipid yield was determined gravimetrically, as described in Equation (1). The lipids isolation process is shown in Figure S14 (bottom).

Lipid extracts were stored at −20 °C until transesterification, after which the resulting FAMEs were analyzed by gas chromatography (GC).

### 3.8. Recovery of Ionic Liquid

Given the economic and environmental implications of ionic liquid synthesis, its recovery and reuse were evaluated to assess the process viability. Two extraction cycles were performed: in the first, freshly synthesized IL was used, and after extraction, it was recovered, purified, and reused in a second extraction cycle under the same conditions. After separation of the aqueous and organic phases, water from the aqueous phase was removed using a rotary evaporator at 40 °C, followed by vacuum drying for 24 h. The resulting mixture, consisting of IL and polar compounds, was washed three times with acetonitrile to dissolve the IL and precipitate the polar fraction. Precipitates were separated by centrifugation (9500 rpm, 3 min) and filtration through 0.22 μm PTFE syringe filters. Acetonitrile was subsequently removed by rotary evaporation (40 °C), leaving the recovered IL, which was further dried under vacuum for 48 h. Purity of the recycled IL was confirmed by ^1^H and ^13^C NMR, DSC, and FTIR, as described previously.

### 3.9. Transesterification

Following lipid extraction, fatty acids must be converted into fatty acid methyl esters (FAMEs) to enable efficient identification and quantification by gas chromatography. Since fatty acids in biological samples occur bound in lipids, hydrolysis (e.g., saponification) is often required to release them [105]. Free fatty acids are difficult to separate by GC due to their polarity; therefore, derivatization into FAMEs increases volatility, reduces polarity, and improves chromatographic resolution [106,107,108,109]. In this study, the transesterification protocol was adapted from previously reported methodologies [110,111,112,113]. Lipid extracts (~10 mg) were subjected to transesterification prior to fatty acid analysis. Each sample was mixed with 1.5 mL of 0.5 M NaOH in methanol and 2 μL of tetradecane (C14:0, internal standard), then heated at 65 °C for 10 min. After cooling, 2 mL of BF_3_–methanol solution (14%) was added, and the mixture was reheated at 65 °C for 20 min. The resulting FAMEs were extracted with 2 mL hexane, followed by phase separation using 2 mL of an aqueous solution of 20% NaCl. This extraction step with hexane was repeated three times, and the combined organic phases were evaporated at 40 °C under reduced pressure. The residue was resuspended in 1 mL hexane p.a., spiked with 2 μL nonane (C9:0, internal standard), filtered through a 0.22 μm PTFE syringe filter, and transferred to 2 mL GC vials for analysis.

### 3.10. Fatty Acid Methyl Esters (FAMEs) Composition Analysis

Following transesterification, the fatty acid profile of the extracted lipids was determined by GC. Nonane (C9:0) was used as the internal standard. GC analyses were performed on a 6890 system (Agilent Technologies) equipped with a flame ionization detector (FID) and an Agilent J&W VF-5ms column (length: 30 m, ID: 0.25 mm and film: 0.25 μm thickness). The injector was maintained at 250 °C, operating with a split ratio of 1:10. The oven program consisted of an initial temperature of 40 °C (1 min), followed by heating at 10 °C/min to 150 °C (15 min), then 5 °C/min to 250 °C, and finally 10 °C/min to 320 °C (15 min). Helium was used as the carrier gas at 1.0 mL/min. Data acquisition was carried out with Clarity software (v.7.0.00.258). For identification and quantification, a Supelco 37 Component FAME Mix (CRM47885) was employed. Response factors (RFs) were calculated to quantify FAMEs. Peaks not included in the standard mix were further characterized by gas chromatography with mass spectrometry (GC–MS). Analyses were performed by using gas chromatography (6890, Agilent Technologies), coupled to a mass spectrometer (5973 GC/MSD, Agilent Technologies) with the same column and temperature program as described above. Samples (1 μL) were injected with a split ratio of 1:30 (30.0 mL/min split flow), using helium as the carrier gas at a constant flow rate of 1.0 mL/min. The inlet was maintained at 250 °C. Mass spectra were acquired in electron impact mode (70 eV), operating in EMV mode at 1259 V, with a scan range of m/z 45–600. Data was processed with MSD ChemStation software, (E.0202 1431) and compound identification was confirmed by comparison with the NIST (National Institute of Standards and Technology) mass spectral library.

### 3.11. Statistical Analysis

Graphpad Prism 8 was used for statistical analysis. Statistical significant differences were calculated through One-Way Analysis of Variance (ANOVA) by Tukey’s multiple comparisons tests and a *p*-value < 0.05 was considered significant. Data is expressed as average ± standard deviation from at least three independent experiments.

## 4. Conclusions

This work highlights the potential of integrating microwave-assisted pretreatment with choline-derived ionic liquid ([N_1 1 2OH 2OH_][C_6_H_11_O_2_]) for the efficient extraction of lipids from *Chlorella vulgaris*. By combining IL pretreatment with an environmentally friendly solvent system, it was possible to obtain lipid and FAME yields comparable to or higher than those obtained with conventional extraction methods, while avoiding the use of conventional organic solvents such as chloroform. Moreover, using the ionic liquid as pretreatment allowed the valorization of the supernatant phase, a phase that is scarcely explored in the literature, yet considerably rich in lipids (10.61 ± 0.39% from the total of 14.29 ± 0.75% obtained from 1:8 (*m*/*m*) ratio under 5 min at 750 W and 60 °C) and FAMEs (24.46 ± 0.48 mg/g algae). This represents an advantage when compared to the conventional methods, also enabling greater lipid extraction (14.29% vs. 2.61%–4.53% for the other methods). Although the extraction performed by Soxhlet with chloroform and methanol led to the best lipid extraction yield (13.04 ± 0.16%), it is worth noting that this method is conducted under extreme conditions and using hazardous solvents.

Furthermore, the IL approach herein explored also proved to be advantageous in terms of recyclability, allowing for its reuse, and representing a more sustainable alternative to toxic imidazolium-based ILs. In addition, the use of ethyl acetate/ethanol (1:1 *v*/*v*) as a more environmentally friendly substitute for chloroform further improves the environmental profile of the method. Overall, this study provides evidence that the MAE–IL applied strategy not only improves lipid accessibility through effective cell wall disruption but also offers a more environmentally friendly route for the valorization of microalgal bioproducts. However, despite these clear benefits, the multi-step nature of the process may increase operational complexity and overall costs, potentially limiting its large-scale implementation. Future work should be focused on scaling up the process, assessing economic feasibility, and optimizing the IL design to further minimize environmental impact, simplify the workflow, and potentially enable its safe incorporation into the final product, thereby opening avenues for food or medicinal applications.

## Figures and Tables

**Table 1:** FAME composition (mg/g algae and wt%) obtained from *C. vulgaris* using conventional methods. (n.d.—non detected and * standard deviation < 0.01 (mg/g algae)).

FAMEs	Soxhlet CHCl_3_:MeOH (2:1)	Bligh & Dyer	Soxhlet *n*-Hexane	Folch
**C4:0**	0.04 * (0.09%)	0.03 * (0.17%)	0.01 * (0.11%)	0.01 * (0.17%)
**C6:0**	0.03 * (0.07%)	0.01 * (0.06%)	0.01 * (0.22%)	n.d.
**C10:0**	0.12 * (0.29%)	0.04 * (0.23%)	0.01 * (0.18%)	0.02 * (0.32%)
**C12:0**	0.06 * (0.14%)	0.01 * (0.05%)	0.01 * (0.20%)	n.d.
**C14:0**	0.25 ± 0.02 (0.58%)	0.07 ± 0.01 (0.39%)	0.05 * (0.94%)	0.02 * (0.35%)
**C15:0**	0.17 ± 0.01 (0.39%)	0.06 * (0.33%)	0.03 * (0.48%)	0.01 * (0.27%)
**C16:2 trans**	2.76 ± 0.12 (6.41%)	1.06 ± 0.01 (6.08%)	0.10 * (1.71%)	0.34 ± 0.03 (6.18%)
**C16:3 trans**	6.35 ± 0.08 (14.74%)	2.60 ± 0.10 (14.94%)	0.25 ± 0.01 (4.49%)	0.85 ± 0.04 (15.42%)
**C16:1 cis**	0.91 ± 0.03 (2.10%)	0.38 ± 0.03 (2.19%)	0.07 * (1.18%)	0.13 * (2.37%)
**C16:1 trans**	0.34 ± 0.02 (0.79%)	0.13 ± 0.01 (0.75%)	0.02 * (0.42%)	0.04 * (0.78%)
**C16:0**	7.90 ± 0.07 (18.33%)	2.97 ± 0.15 (17.05%)	1.34 ± 0.07 (23.82%)	0.91 ± 0.02 (16.63%)
**15-Methylpalmitic acid**	1.11 ± 0.05 (2.57%)	0.41 ± 0.01 (2.35%)	0.04 * (0.77%)	0.12 ± 0.01 (2.22%)
**Mix of compounds to C17 derived FAMEs**	2.81 ± 0.15 (6.51%)	1.27 ± 0.08 (7.30%)	0.98 ± 0.08 (17.43%)	0.37 ± 0.02 (6.65%)
**Cascarillic acid**	0.16 ± 0.01 (0.38%)	0.06 * (0.34%)	0.01 * (0.18%)	0.02 * (0.38%)
**C17:3 cis**	0.31 ± 0.01 (0.72%)	0.11 ± 0.01 (0.61%)	0.01 * (0.09%)	0.03 * (0.57%)
**C17:0**	0.46 ± 0.04 (1.06%)	0.18 ± 0.02 (1.06%)	0.03 * (0.46%)	0.05 * (0.93%)
**C18:2 cis**	5.95 ± 0.14 (13.81%)	2.55 ± 0.17 (14.64%)	0.23 * (4.15%)	0.75 ± 0.06 (13.67%)
**C18:3 trans + C18:1 cis + C18:2 trans**	10.99 ± 0.35 (25.49%)	4.59 ± 0.09 (26.36%)	1.74 ± 0.02 (30.86%)	1.51 ± 0.07 (27.49%)
**C18:1 trans**	0.85 ± 0.04 (1.96%)	0.34 ± 0.03 (1.95%)	0.05 * (0.82%)	0.11 ± 0.01 (2.09%)
**C18:0**	1.00 ± 0.08 (2.33%)	0.42 ± 0.03 (2.40%)	0.52 ± 0.02 (9.26%)	0.14 ± 0.01 (2.47%)
**C20:4**	0.13 ± 0.01 (0.29%)	0.03 * (0.19%)	0.02 * (0.34%)	0.02 * (0.31%)
**C20:5**	0.07 * (0.15%)	0.02 * (0.11%)	0.01 * (0.15%)	0.01 * (0.20%)
**C20:0**	0.13 ± 0.01 (0.31%)	0.03 * (0.17%)	0.03 * (0.59%)	0.01 * (0.24%)
**C24:0**	0.21 ± 0.02 (0.49%)	0.05 * (0.28%)	0.07 * (1.15%)	0.02 * (0.30%)
**Total**	43.10 ± 0.21 (100%)	17.41 ± 0.54 (100%)	5.64 ± 0.19 (100%)	5.49 ± 0.29 (100%)

**Table 2:** FAME composition (mg/g algae and wt%) obtained from the supernatant of *C. vulgaris*. (n.d.—non detected and * standard deviation < 0.01 (mg/g algae)).

FAMEs	w/o IL and w/o MW	w/o IL and w/MW	1:4 (algae/IL)	1:8 (algae/IL)	1:8 (algae/IL) Recycled
**C4:0**	n.d.	0.01 * (0.13%)	0.02 * (0.24%)	0.04 * (0.15%)	0.03 * (0.13%)
**C6:0**	0.01 * (0.29%)	0.02 * (0.34%)	0.06 ± 0.01 (0.84%)	0.12 * (0.48%)	0.11 * (0.44%)
**C10:0**	0.01 * (0.19%)	0.01 * (0.14%)	0.02 * (0.28%)	0.03 * (0.12%)	0.04 * (0.15%)
**C12:0**	0.01 * (0.11%)	0.01 * (0.11%)	0.02 * (0.22%)	0.02 * (0.10%)	0.04 * (0.16%)
**C14:0**	0.04 * (0.79%)	0.03 * (0.55%)	0.06 * (0.85%)	0.14 * (0.56%)	0.15 ± 0.01 (0.61%)
**C15:0**	0.02 * (0.34%)	0.01 * (0.24%)	0.02 * (0.29%)	0.07 * (0.28%)	0.07 * (0.26%)
**C16:2 trans**	0.06 * (1.24%)	0.14 ± 0.01 (2.76%)	0.37 ± 0.03 (4.81%)	1.41 ± 0.03 (5.76%)	1.07 ± 0.03 (4.28%)
**C16:3 trans**	0.18 ± 0.01 (3.71%)	0.19 ± 0.01 (3.78%)	1.02 ± 0.05 (13.40%)	3.31 ± 0.04 (13.51%)	2.89 ± 0.07 (11.52%)
**C16:1 cis**	0.03 * (0.64%)	0.06 ± 0.01 (1.25%)	0.09 ± 0.01 (1.18%)	0.43 ± 0.01 (1.74%)	0.32 ± 0.02 (1.27%)
**C16:1 trans**	0.04 * (0.80%)	0.04 * (0.74%)	0.15 * (1.93%)	0.17 * (0.71%)	0.25 ± 0.01 (0.99%)
**C16:0**	0.92 ± 0.06 (19.14%)	0.88 ± 0.02 (17.44%)	1.39 ± 0.08 (18.31%)	4.24 ± 0.09 (17.34%)	4.48 ± 0.15 (17.84%)
**15-Methylpalmitic acid**	0.04 * (0.84%)	0.04 * (0.73%)	0.15 ± 0.01 (2.01%)	0.53 ± 0.02 (2.17%)	0.45 ± 0.02 (1.78%)
**Mix of compounds to C17 derived FAMEs**	2.44 ± 0.16 (50.62%)	2.52 ± 0.09 (50.12%)	1.02 ± 0.06 (13.43%)	1.84 ± 0.10 (7.51%)	3.41 ± 0.04 (13.58%)
**Cascarillic acid**	0.01 * (0.22%)	0.01 * (0.16%)	0.02 * (0.20%)	0.08 ± 0.01 (0.34%)	0.07 * (0.30%)
**C17:3 cis**	n.d.	0.03 * (0.66%)	0.03 * (0.36%)	0.13 ± 0.01 (0.51%)	0.11 ± 0.01 (0.44%)
**C17:0**	0.02 * (0.38%)	0.02 * (0.42%)	0.05 * (0.65%)	0.20 * (0.83%)	0.17 ± 0.01 (0.66%)
**C18:2 cis**	0.15 ± 0.01 (3.01%)	0.14 ± 0.01 (2.71%)	0.83 ± 0.07 (10.87%)	3.47 ± 0.20 (14.20%)	3.01 ± 0.03 (12.01%)
**C18:3 trans + C18:1 cis + C18:2 trans**	0.24 ± 0.01 (5.07%)	0.24 ± 0.01 (4.79%)	1.64 ± 0.10 (21.61%)	6.73 ± 0.19 (27.50%)	6.19 ± 0.12 (24.68%)
**C18:1 trans**	0.05 * (1.01%)	0.02 * (0.46%)	0.07 * (0.86%)	0.32 * (1.32%)	0.37 ± 0.02 (1.47%)
**C18:0**	0.52 ± 0.03 (10.79%)	0.49 ± 0.02 (9.75%)	0.43 ± 0.01 (5.71%)	0.90 ± 0.01 (3.69%)	1.50 ± 0.07 (6.00%)
**C20:4**	n.d.	0.02 * (0.42%)	0.02 * (0.29%)	0.06 * (0.25%)	0.03 * (0.12%)
**C20:5**	n.d.	0.01 * (0.15%)	0.01 * (0.15%)	0.03 * (0.14%)	0.02 * (0.08%)
**C20:0**	0.01 * (0.11%)	0.06 * (1.25%)	0.05 * (0.62%)	0.04 * (0.16%)	0.14 ± 0.01 (0.57%)
**C24:0**	0.03 * (0.71%)	0.04 * (0.89%)	0.07 * (0.87%)	0.15 ± 0.01 (0.62%)	0.16 ± 0.01 (0.63%)
**Total**	4.82 ± 0.26 (100%)	5.02 ± 0.18 (100%)	7.61 ± 0.30 (100%)	24.46 ± 0.48 (100%)	25.08 ± 0.23 (100%)

**Table 3:** FAME composition (mg/g algae and wt%) obtained from the supernatant and biomass sediment of *C. vulgaris* (* standard deviation < 0.01 (mg/g algae)).

FAMEs	Soxhlet CHCl_3_:MeOH (2:1)	Bligh & Dyer	AcEt/EtOH (1:1)	Supernatant w/o IL and w/MW	Biomass Sediment w/o IL and w/MW	Extraction Total w/o IL and w/MW	Supernatant 1:8 (algae/IL)	Biomass Sediment 1:8 (algae/IL)	Extraction Total 1:8 (algae/IL)
**C4:0**	0.04 * (0.09%)	0.03 * (0.17%)	0.01 * (0.08%)	0.01 * (0.13%)	0.01 * (0.07%)	0.02 * (0.09%)	0.04 * (0.15%)	0.02 * (0.09%)	0.05 * (0.12%)
**C6:0**	0.03 * (0.07%)	0.01 * (0.06%)	0.01 * (0.06%)	0.02 * (0.34%)	0.01 * (0.08%)	0.03 * (0.14%)	0.12 * (0.48%)	0.02 * (0.08%)	0.13 * (0.31%)
**C10:0**	0.12 * (0.29%)	0.04 * (0.23%)	0.03 * (0.16%)	0.01 * (0.14%)	0.02 * (0.11%)	0.03 * (0.12%)	0.03 * (0.12%)	0.02 * (0.13%)	0.05 * (0.12%)
**C12:0**	0.06 * (0.14%)	0.01 * (0.05%)	0.01 * (0.07%)	0.01 * (0.11%)	0.01 * (0.06%)	0.02 * (0.07%)	0.02 * (0.10%)	0.01 * (0.07%)	0.04 * (0.09%)
**C14:0**	0.25 ± 0.02 (0.58%)	0.07 ± 0.01 (0.39%)	0.07 * (0.46%)	0.03 * (0.55%)	0.11 ± 0.01 (0.64%)	0.13 ± 0.01 (0.62%)	0.14 * (0.56%)	0.09 *(0.49%)	0.23 (0.53%)
**C15:0**	0.17 ± 0.01 (0.39%)	0.06 * (0.33%)	0.06 * (0.36%)	0.01 * (0.24%)	0.07 ± 0.01 (0.44%)	0.08 ± 0.01 (0.39%)	0.07 * (0.28%)	0.06 * (0.32%)	0.13 (0.30%)
**C16:2 trans**	2.76 ± 0.12 (6.41%)	1.06 ± 0.01 (6.08%)	0.90 ± 0.07 (5.76%)	0.14 ± 0.01 (2.76%)	0.77 ± 0.05 (4.69%)	0.90 ± 0.04 (4.23%)	1.41 ± 0.03 (5.76%)	1.16 ± 0.07 (6.43%)	2.57 ± 0.06 (6.05%)
**C16:3 trans**	6.35 ± 0.08 (14.74%)	2.60 ± 0.10 (14.94%)	2.00 ± 0.14 (12.74%)	0.19 ± 0.01 (3.78%)	1.99 ± 0.14 (12.19%)	2.02 ± 0.14 (9.45%)	3.31 ± 0.04 (13.51%)	2.65 ± 0.08 (14.63%)	5.95 ± 0.11 (13.99%)
**C16:1 cis**	0.91 ± 0.03 (2.10%)	0.38 ± 0.03 (2.19%)	0.31 ± 0.02 (1.98%)	0.06 ± 0.01 (1.25%)	0.28 * (1.70%)	0.34 * (1.59%)	0.43 ± 0.01 (1.74%)	0.36 ± 0.01 (1.99%)	0.79 ± 0.02 (1.85%)
**C16:1 trans**	0.34 ± 0.02 (0.79%)	0.13 ± 0.01 (0.75%)	0.12 ± 0.01 (0.76%)	0.04 * (0.74%)	0.02 * (0.10%)	0.05 * (0.25%)	0.17 * (0.71%)	0.17 ± 0.01 (0.95%)	0.35 ± 0.01 (0.82%)
**C16:0**	7.90 ± 0.07 (18.33%)	2.97 ± 0.15 (17.05%)	2.92 ± 0.21 (18.56%)	0.88 ± 0.02 (17.44%)	4.19 ± 0.37 (25.64%)	5.06 ± 0.40 (23.67%)	4.24 ± 0.09 (17.34%)	2.87 ± 0.12 (15.89%)	7.12 ± 0.19 (16.72%)
**15-Methylpalmitic acid**	1.11 ± 0.05 (2.57%)	0.41 ± 0.01 (2.35%)	0.39 ± 0.02 (2.51%)	0.04 * (0.73%)	0.51 ± 0.05 (3.14%)	0.55 ± 0.05 (2.57%)	0.53 ± 0.02 (2.17%)	0.38 ± 0.03 (2.12%)	0.91 ± 0.04 (2.15%)
**Mix of compounds to C17 derived FAMEs**	2.81 ± 0.15 (6.51%)	1.27 ± 0.08 (7.30%)	1.50 ± 0.13 (9.53%)	2.52 ± 0.09 (50.12%)	1.37 ± 0.11 (8.40%)	4.09 ± 0.22 (19.11%)	1.84 ± 0.10 (7.51%)	1.71 ± 0.04 (9.47%)	3.55 ± 0.14 (8.35%)
**Cascarillic acid**	0.16 ± 0.01 (0.38%)	0.06 * (0.34%)	0.03 * (0.18%)	0.01 * (0.16%)	0.02 * (0.12%)	0.03 * (0.13%)	0.08 ± 0.01 (0.34%)	0.07 * (0.37%)	0.15 ± 0.01 (0.35%)
**C17:3 cis**	0.31 ± 0.01 (0.72%)	0.11 ± 0.01 (0.61%)	0.01 * (0.08%)	0.03 * (0.66%)	0.06 * (0.39%)	0.10 * (0.45%)	0.13 ± 0.01 (0.51%)	0.01 * (0.07%)	0.14 ± 0.01 (0.32%)
**C17:0**	0.46 ± 0.04 (1.06%)	0.18 ± 0.02 (1.06%)	0.17 ± 0.01 (1.07%)	0.02 * (0.42%)	0.22 ± 0.02 (1.33%)	0.24 ± 0.02 (1.11%)	0.20 * (0.83%)	0.17 * (0.91%)	0.37 * (0.87%)
**C18:2 cis**	5.95 ± 0.14 (13.81%)	2.55 ± 0.17 (14.64%)	2.22 ± 0.07 (14.14%)	0.14 ± 0.01 (2.71%)	1.93 ± 0.13 (11.80%)	2.06 ± 0.13 (9.64%)	3.47 ± 0.20 (14.20%)	2.37 ± 0.07 (13.12%)	5.85 ± 0.23 (13.74%)
**C18:3 trans + C18:1 cis + C18:2 trans**	10.99 ± 0.35 (25.49%)	4.59 ± 0.09 (26.36%)	3.91 ± 0.14 (24.87%)	0.24 ± 0.01 (4.79%)	3.53 ± 0.25 (21.61%)	3.77 ± 0.26 (17.62%)	6.73 ± 0.19 (27.50%)	4.72 ± 0.16 (26.11%)	11.45 ± 0.34 (26.91%)
**C18:1 trans**	0.85 ± 0.04 (1.96%)	0.34 ± 0.03 (1.95%)	0.34 ± 0.02 (2.14%)	0.02 * (0.46%)	0.28 ± 0.01 (1.74%)	0.31 ± 0.01 (1.44%)	0.32 * (1.32%)	0.39 ± 0.02 (2.15%)	0.71 ± 0.02 (1.67%)
**C18:0**	1.00 ± 0.08 (2.33%)	0.42 ± 0.03 (2.40%)	0.52 ± 0.03 (3.31%)	0.49 ± 0.02 (9.75%)	0.71 ± 0.03 (4.32%)	1.20 ± 0.05 (5.59%)	0.90 ± 0.01 (3.69%)	0.63 ± 0.05 (3.51%)	1.54 ± 0.04 (3.61%)
**C20:4**	0.13 ± 0.01 (0.29%)	0.03 * (0.19%)	0.08 * (0.52%)	0.02 * (0.42%)	0.10 ± 0.01 (0.60%)	0.12 ± 0.01 (0.56%)	0.06 * (0.25%)	0.07 * (0.37%)	0.13 ± 0.01 (0.30%)
**C20:5**	0.07 * (0.15%)	0.02 * (0.11%)	0.02 * (0.12%)	0.01 * (0.15%)	0.04 * (0.22%)	0.04 * (0.21%)	0.03 * (0.14%)	0.02 * (0.10%)	0.05 * (0.12%)
**C20:0**	0.13 ± 0.01 (0.31%)	0.03 * (0.17%)	0.04 * (0.27%)	0.06 * (1.25%)	0.05 * (0.29%)	0.11 * (0.52%)	0.04 * (0.16%)	0.06 * (0.32%)	0.10 ± 0.01 (0.23%)
**C24:0**	0.21 ± 0.02 (0.49%)	0.05 * (0.28%)	0.04 * (0.27%)	0.04 * (0.89%)	0.05 * (0.30%)	0.09 ± 0.01 (0.44%)	0.15 ± 0.01 (0.62%)	0.06 * (0.30%)	0.21 ± 0.01 (0.48%)
**Total**	43.10 ± 0.21 (100%)	17.41 ± 0.54 (100%)	15.71 ± 0.61 (100%)	5.02 ± 0.18 (100%)	16.32 ± 0.45 (100%)	21.39 ± 0.58 (100%)	24.46 ± 0.48 (100%)	18.09 ± 0.35 (100%)	42.56 ± 0.64 (100%)

## Data Availability

Data is contained within the article or Supplementary Materials.

## Supplementary Materials

The following supporting information can be downloaded at: https://www.mdpi.com/article/10.3390/molecules30234611/s1, Figures S1–S4: Characterization of IL (^1^H and ^13^C NMR, FTIR-ATR and DSC); Figure S5: Effect of supernatant stirring time with n-hexane on lipid extraction yield; Figure S6: Effect of Microwave Pretreatment and Ionic Liquid Ratio on Lipid Recovery from *C. vulgaris* (supernatant); Figure S7: DSC Thermogram of pure and recovered IL; Figure S8: FTIR-ATR spectra of IL pure and recovered after extraction; Figure S9: FTIR-ATR spectra of pure IL and lipid extracts obtained; Figure S10: FAME yield from *C. vulgaris* supernatant under different tested conditions; Figure S11: FAME yield from supernatant and biomass sediment of *C. vulgaris* after MW pretreatment, with and without IL; Figure S12: Total FAME yield from *C. vulgaris* obtained by MW pretreatment with and without IL, conventional methods, and direct extraction using ethyl acetate/ethanol (1:1); Figures S13 and S14: Illustrative representations of lipid extraction processes; Figure S15: Chemical structure of IL [N_1 1 2OH 2OH_][C_6_H_11_O_2_]. Table S1. Comparison of the lipids and FAMEs yields obtained from the different techniques: IL 1:8 (algae:IL) + MW, MW without IL, Bligh and Dyer, Folch, Soxhlet chloroform:methanol (2:1), Soxhlet nhexane and ethyl acetate:ethanol (1:1). References [114,115] are cited in Supplementary Materials.
