# Supplementary material for: From Biomass to Efficient Lipid Recovery: Choline-Based Ionic Liquids and Microwave Extraction of Chlorella vulgaris
Source: Molecules. 2025 Dec 1;30(23):4611. doi: 10.3390/molecules30234611 (PMC12693379; doi:10.3390/molecules30234611)
Supplement: Supplementary file 1 [file molecules-30-04611-s001.zip › molecules-3948016-supplementary.pdf]

From Biomass to Efficient Lipid Recovery: Choline-Based Ionic Liquids and Microwave Extraction of *Chlorella vulgaris*

Daniela A. S. Agostinho[1], Andreia F. M. Santos[1], José M. S. S. Esperança[1], Patrícia M. Reis[1], Ana Rita C. Duarte[1,*] and Márcia G. Ventura[1,2,*]

[1] Laboratório Associado para a Química Verde e Tecnologias Sustentáveis-Rede de Química e Tecnologia (LAQV-REQUIMTE), Departamento de Química, NOVA School of Science and Technology, Universidade NOVA de Lisboa, Campus da Caparica, 2829-516 Caparica, Portugal; d.agostinho@campus.fct.unl.pt (D.A.S.A.); preis@fct.unl.pt (P.M.R.)
[2] Escola Superior de Tecnologia do Barreiro, Instituto Politécnico de Setúbal (ESTB/IPS), 2839-001 Lavradio, Portugal
* Correspondence: ard08968@fct.unl.pt (A.R.C.D.); marcia.ventura@estbarreiro.ips.pt (M.G.V.)

Characterization of IL

NMR Results

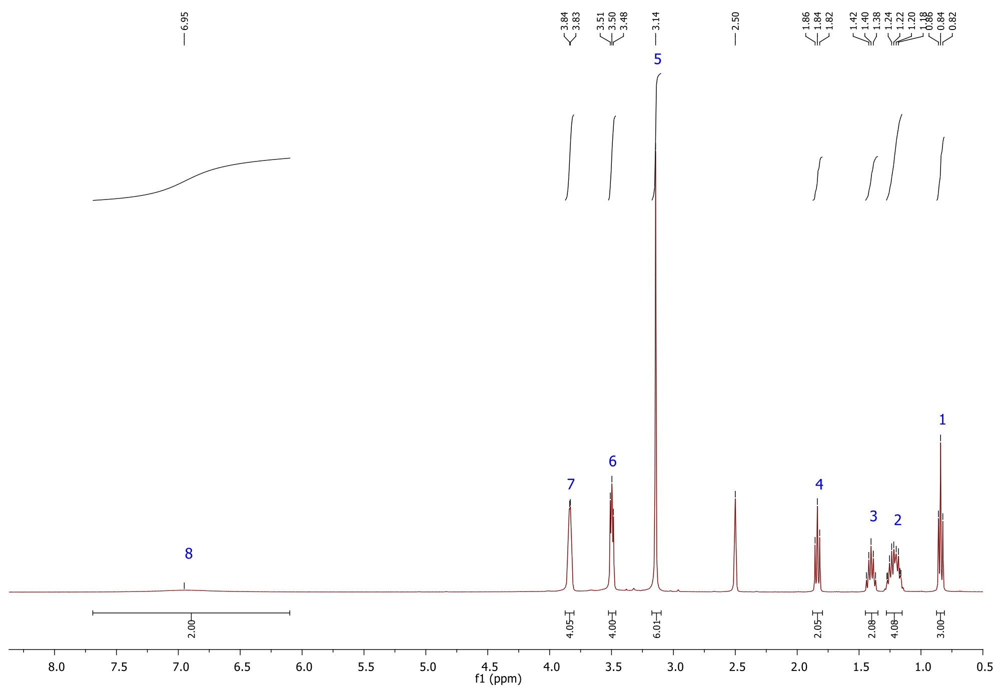

Figure S1 - ^{1}H NMR of $[N_{1\,1\,2OH\,2OH}][C_6H_{11}O_2]$.

^{1}H NMR (DMSO-*d6*, 400 MHz): δ = 0.84 (t, 3H, *J*= 8Hz, C**H**$_3$CH$_2$CH$_2$CH$_2$CH$_2$COO), 1.16-1.27 (m, 4H, CH$_3$C**H$_2$**C**H$_2$**CH$_2$CH$_2$COO), 1.40 (qt, 2H, *J*= 7Hz, CH$_3$CH$_2$CH$_2$C**H$_2$**CH$_2$COO), 1.84 (t, 2H, *J*=

8Hz, $CH_3CH_2CH_2CH_2C\mathbf{H}_2COO$), 3.14 (s, 6H, N($C\mathbf{H}_3$)$_2$), 3.50 (t, 4H, *J*= 6Hz, N($C\mathbf{H}_2CH_2OH$)$_2$), 3.83-3.84 (m, 4H, N($CH_2C\mathbf{H}_2OH$)$_2$), 6.95 (m, 2H, N($CH_2CH_2O\mathbf{H}$)$_2$) ppm

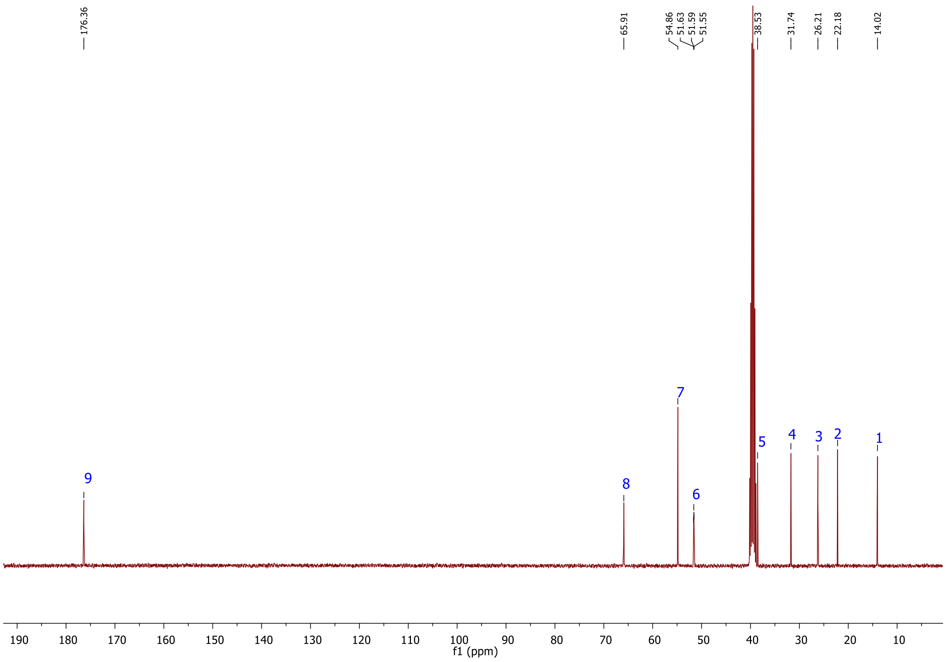

Figure S2 – ^{13}C NMR of [$N_{1\,1\,2OH\,2OH}$][$C_6H_{11}O_2$].

^{13}C NMR (DMSO-*d6*, 100 MHz): δ = 14.02 ($\underline{C}H_3CH_2CH_2CH_2CH_2COO$), 22.18 ($CH_3\underline{C}H_2CH_2CH_2CH_2COO$), 26.21 ($CH_3CH_2CH_2\underline{C}H_2CH_2COO$), 31.74 ($CH_3CH_2\underline{C}H_2CH_2CH_2COO$), 38.53 ($CH_3CH_2CH_2CH_2\underline{C}H_2COO$), 51.59 (N($\underline{C}H_3$)$_2$), 54.86 (N($CH_2\underline{C}H_2OH$)$_2$), 65.91 (N($\underline{C}H_2CH_2OH$)$_2$), 176.36 ($CH_3CH_2CH_2CH_2CH_2\underline{C}OO$) ppm

FTIR-ATR Results

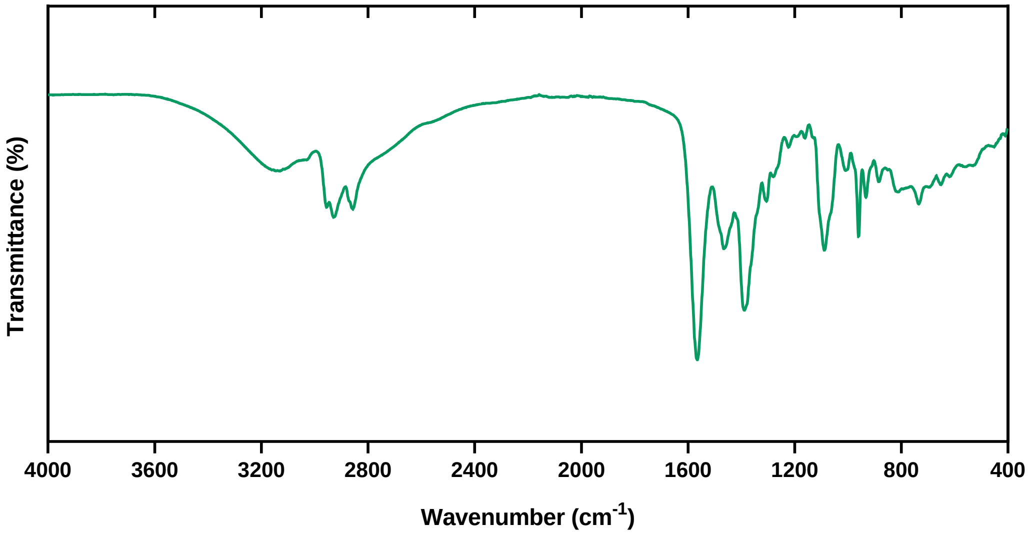

Figure S3 - FTIR spectrum of [$N_{1\,1\,2OH\,2OH}$][$C_6H_{11}O_2$].

Between 3600 cm^{-1} and 3080 cm^{-1}, a broad absorption band is observed, corresponding to the stretching vibrations of hydroxyl (OH) groups, with a more intense peak centered around 3200 cm^{-1}. The region between 3040 and 2770 cm^{-1}, featuring distinct peaks at 3032, 2954, 2928, and 2857 cm^{-1}, as well as a peak at 731 cm^{-1} is attributed to C-H stretching and bending vibrations. The N^+–C (NCH_3) bond is identified by peaks at 1477, and 883 cm^{-1}. A well-defined absorption between 1700 and 1500 cm^{-1} corresponds to the carbonyl (C=O) stretching. Peaks at 1463 and 1388 cm^{-1} are associated with the -CH_2CH_3 group, while the peak at approximately 1396 cm^{-1} likely indicates the presence of carboxylate (COO^-) stretch. Additionally, the peaks at 1304 and 1159 cm^{-1}, as well as at 1086, 1064, 959, and 932 cm^{-1}, are attributed to C-O(H) stretching vibrations [96-101,114,115].

DSC Results

The IL [$N_{1\ 1\ 2OH\ 2OH}$][$C_6H_{11}O_2$], which remains liquid at room temperature and, therefore, is classified as a room temperature ionic liquid, exhibited only a glass transition temperature (T_g) at -80.07 °C. The corresponding thermogram from the second heating run is presented in the figure. No additional thermal events were observed in the DSC analysis.

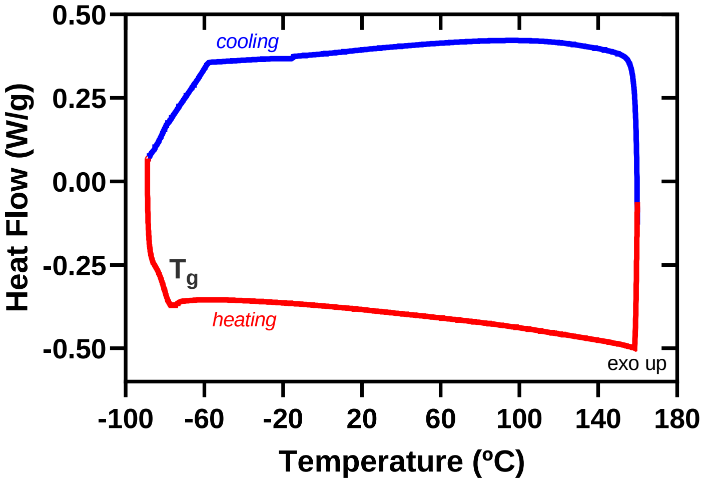

Figure S4 - DSC thermogram of [$N_{1\,1\,2OH\,2OH}$][$C_6H_{11}O_2$]. Second heating (red) and second cooling (blue) runs.

Effect of Supernatant Stirring Time with *n*-hexane on Lipid Extraction Yield

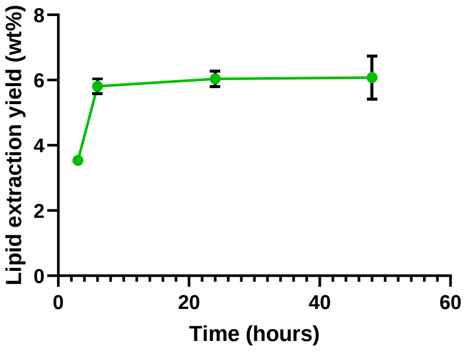

Figure S5. Effect of vigorous stirring time of the supernatant solution in contact with n-hexane on lipid extraction yield. Results are expressed as a percentage of the total mass of algae used. Data are expressed as the mean ± standard deviation of three independent experiments.

Effect of Microwave Pretreatment and Ionic Liquid Ratio on Lipid Recovery from *Chlorella vulgaris* (supernatant)

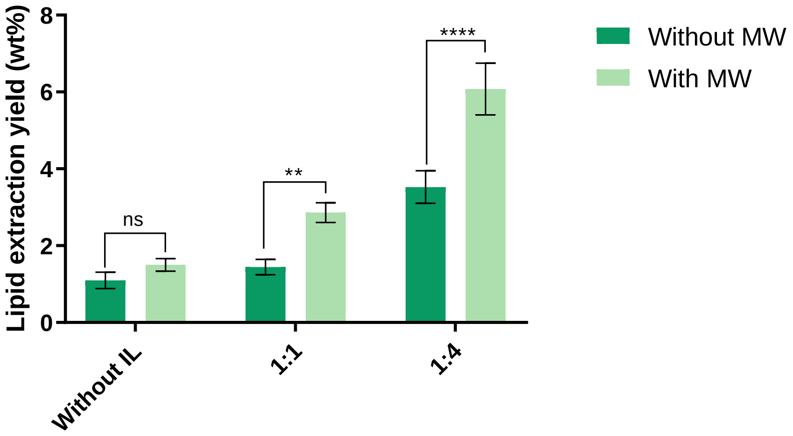

Figure S6. Comparison of lipid content in supernatant, expressed as a percentage of the total mass of algae used for pre-treatment with and without MW pretreatment, both in the absence and presence of IL, at mass ratios of 1:1 and 1:4 (algae:IL). Results are expressed as a percentage of the total mass of algae used. Data are expressed as the mean ± standard deviation of three independent experiments. Statistically significant differences are represented by asterisks: ** $p = 0.0051$, **** $p < 0.0001$ and ns – non-significant.

DSC Thermogram of Pure and Recovered IL

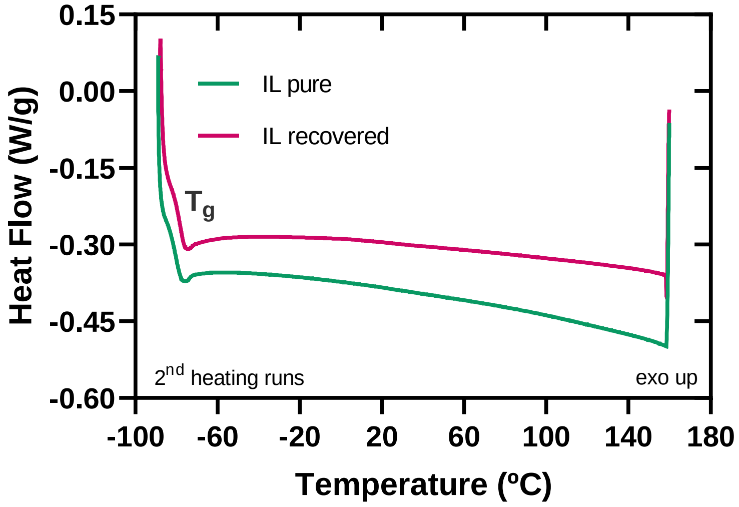

Figure S7. DSC thermograms (second heating run) of pure and recovered $[N_{1\ 1\ 2OH\ 2OH}][C_6H_{11}O_2]$ from extraction with mass ratio 1:8 (algae:IL).

FTIR-ATR Spectra of Pure and Recovered IL After Extraction.

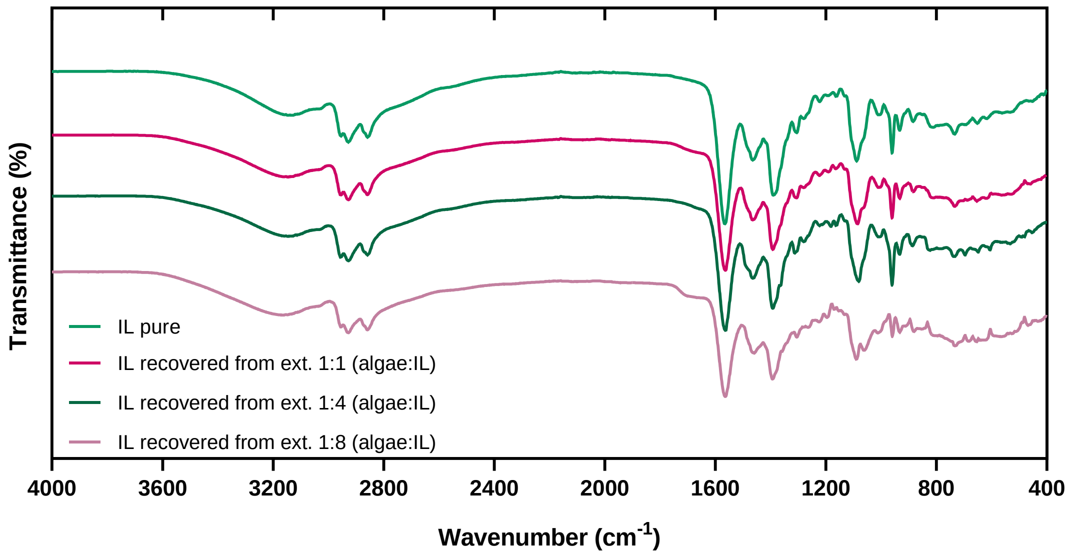

Figure S8. FTIR-ATR spectra of pure and recovered [$N_{1\,1\,2OH\,2OH}$][$C_6H_{11}O_2$] from extraction with mass ratio 1:1, 1:4 and 1:8 (algae:IL).

FTIR-ATR Spectra of Pure IL and Lipid Extracts Obtained.

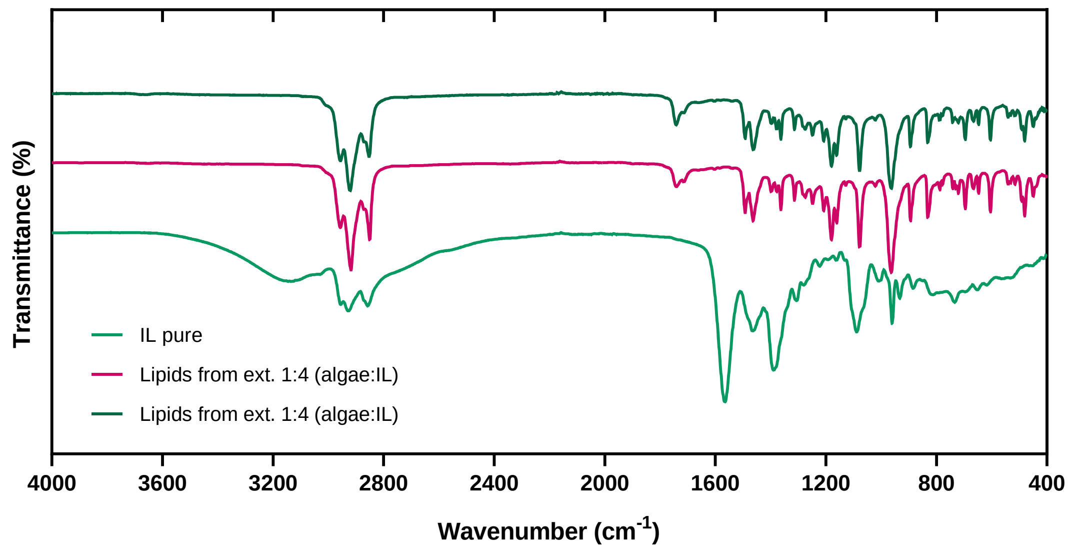

Figure S9. FTIR-ATR spectra of pure [$N_{1\,1\,2OH\,2OH}$][$C_6H_{11}O_2$] and the lipid extract obtained from extractions with a mass ratio of 1:4 and 1:8 (algae:IL).

FAMEs Yield from *C. vulgaris* Supernatant Under Different Tested Conditions.

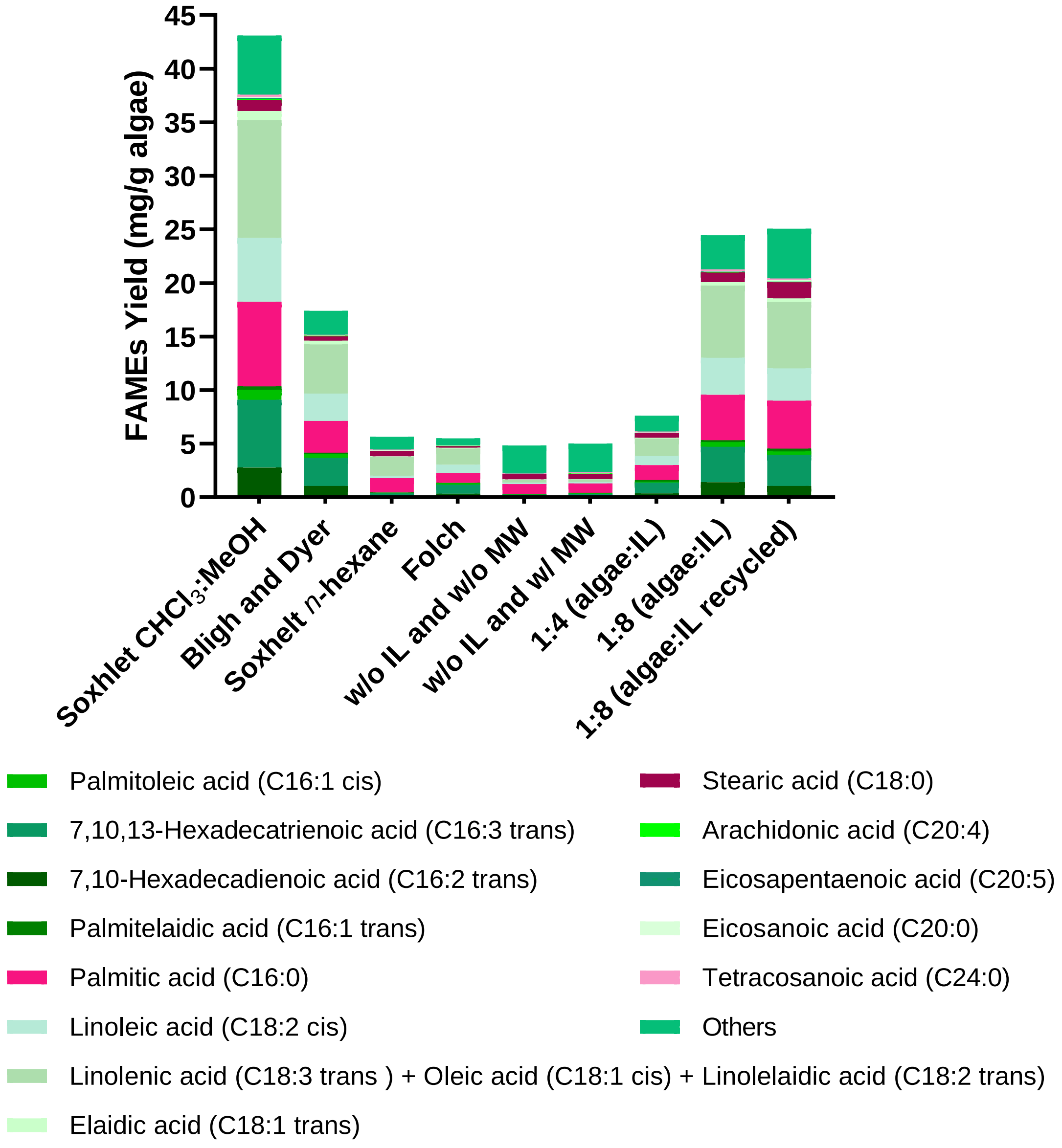

Figure S10. Comparison of FAMEs yield (mg/g algae) obtained under different tested conditions from *C. vulgaris* (supernatant). Data are expressed as the mean ± standard deviation of three independent experiments.

FAMEs Yield from Supernatant and Biomass Sediment of *C. vulgaris* After MW Pretreatment, with and without IL.

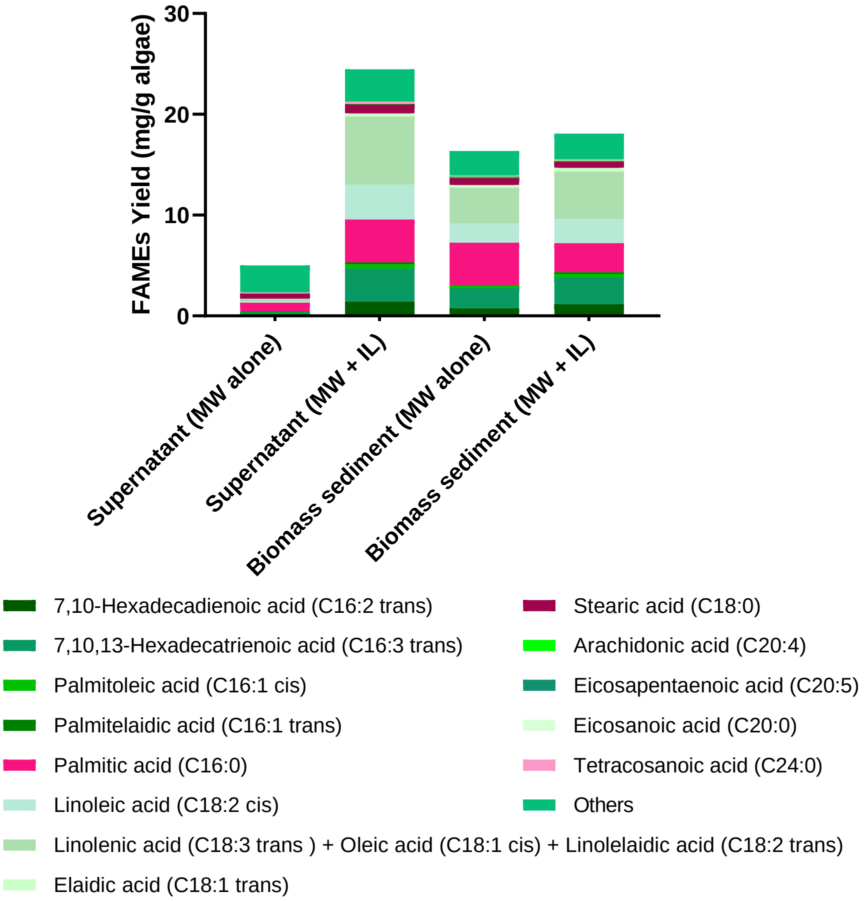

Figure S11. Comparison of the FAMEs yield obtained from the supernatant and biomass sediment in the MW treatment of *C. vulgaris*, with and without IL. Data are presented as the mean ± standard deviation of three independent experiments.

Total FAMEs Yield from *C. vulgaris* Obtained by MW Pretreatment with and without IL, Conventional Methods and Direct Extraction Using Ethyl Acetate:Ethanol (1:1).

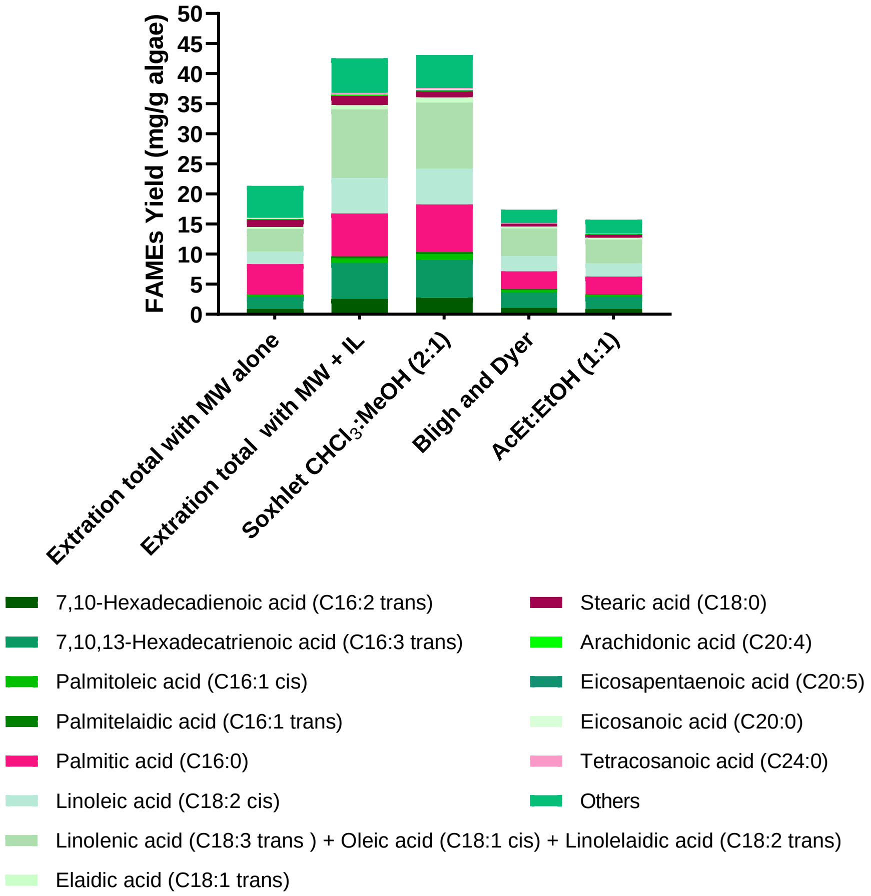

Figure S12. Total FAME yield obtained from *C. vulgaris* through pre-treatment with MW, pre-treatment with MW combined with IL, conventional methods, and direct extraction (without pre-treatment) using an ethyl acetate:ethanol (1:1) mixture. Data are presented as the mean ± standard deviation of three independent experiments.

Table Summarizing Lipids and FAMEs Yield Obtained by Different Methods

Table S1. Comparison of the lipids and FAMEs yields obtained from the different techniques: IL 1:8 (algae:IL) + MW, MW without IL, Bligh and Dyer, Folch, Soxhlet chloroform:methanol (2:1), Soxhlet *n*-hexane and ethyl acetate:ethanol (1:1).

Extraction method	Supernatant		Biomass sediment		Total	
	Lipids Yield (mg/g algae)	FAMEs Yield (mg/g algae)	Lipids Yield (mg/g algae)	FAMEs Yield (mg/g algae)	Lipids Yield (mg/g algae)	FAMEs Yield (mg/g algae)
1:8 (algae:IL) + MW	106.06 ± 3.85	24.46 ± 0.48	36.89 ± 3.98	18.09 ± 0.35	142.94 ± 7.49	42.56 ± 0.64
With MW without IL	15.00 ± 1.35	5.02 ± 0.18	31.25 ± 2.52	16.32 ± 0.45	46.25 ± 3.27	21.39 ± 0.58
Bligh & Dyer					45.28 ± 6.53	17.41 ± 0.54
Folch					26.14 ± 2.15	5.49 ± 0.29
Soxhlet $CHCl_3$:MeOH (2:1)					130.43 ± 1.65	43.10 ± 0.21
Soxhelt *n*-hexane					27.28 ± 5.93	5.64 ± 0.19
AcEt:EtOH (1:1)					44.57 ± 3.42	15.71 ± 0.61

Illustrative Representations of Lipid Extraction Processes.

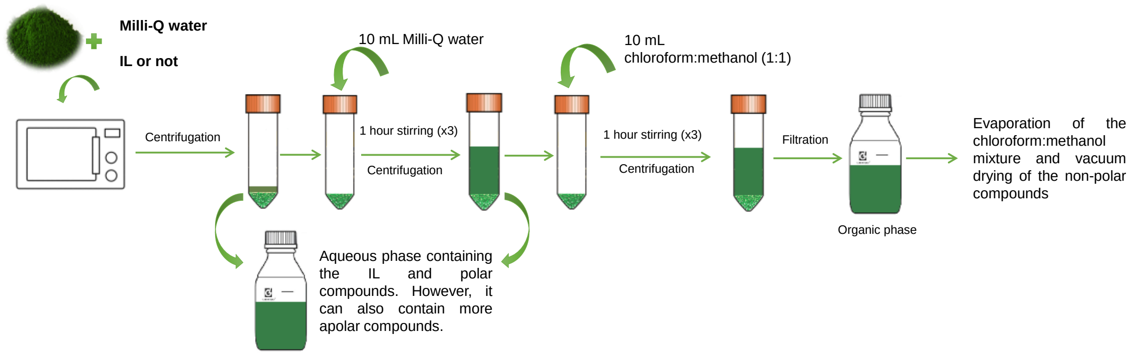

Figure S13. Diagram illustrating the lipids isolation process from the biomass sediment (using chloroform:methanol (1:1, v/v) as the solvent) after pretreatment under optimized conditions.

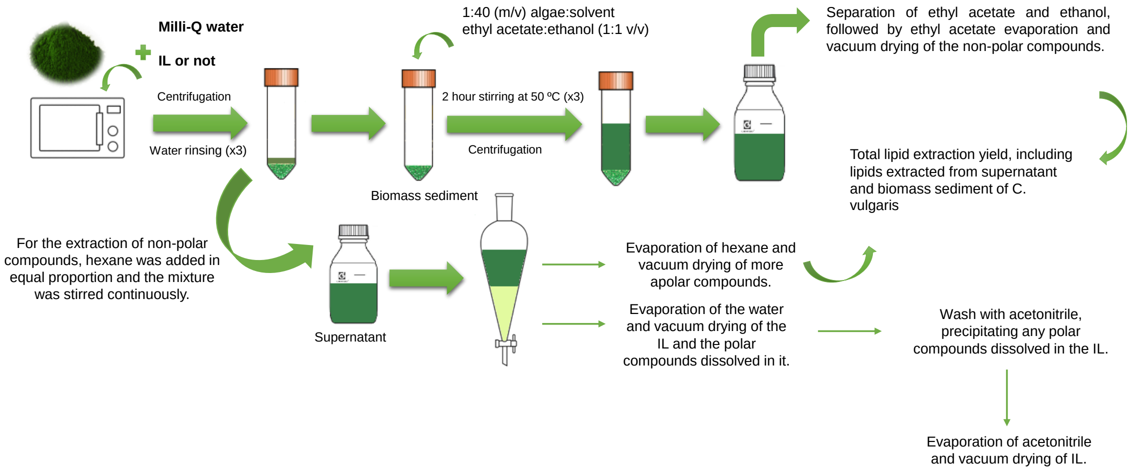

Figure S14. Diagram illustrating the lipids isolation process from the supernatant (using *n*-hexane as the solvent) and from the biomass sediment (using ethyl acetate:ethanol (1:1, v/v) as the solvent) after pretreatment under optimized conditions.

Chemical Structure of IL $[N_{1\,1\,2OH\,2OH}][C_6H_{11}O_2]$

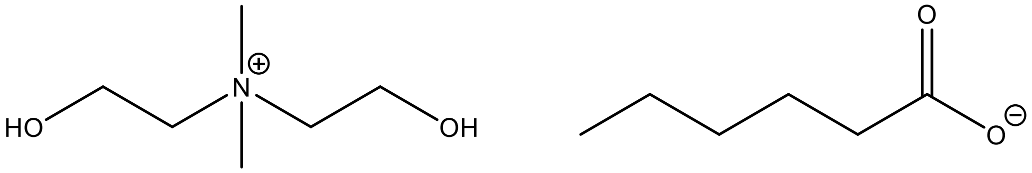

Figure S15. Chemical structure of IL $[N_{1\,1\,2OH\,2OH}][C_6H_{11}O_2]$ synthesized in this work

References

96. Filoda, P.F.; Fetter, L.F.; Fornasier, F.; Schneider, R. de C. de S.; Helfer, G.A.; Tischer, B.; Teichmann, A.; da Costa, A. Ben Fast Methodology for Identification of Olive Oil Adulterated with a Mix of Different Vegetable Oils. *Food Anal. Methods* **2019**, *12*, 293–304, doi:10.1007/s12161-018-1360-5.
97. Zhang, M.; Zhou, Q.; He, J.; Xie, Y.; Yang, H.; He, W. Preparation and Characterization of Ionic Conductive Eutectogels Based on Polyacrylamide Copolymers with Long Hydrophobic Chain . *Chinese J. Chem. Phys.* **2024**, *37*, 685–695, doi:10.1063/1674-0068/cjcp2304039.
98. Hernández-Martínez, M.; Gallardo-Velázquez, T.; Osorio-Revilla, G.; Almaraz-Abarca, N.; Ponce-Mendoza, A.; Vásquez-Murrieta, M.S. Prediction of Total Fat, Fatty Acid Composition and Nutritional Parameters in Fish Fillets Using MID-FTIR Spectroscopy and Chemometrics. *LWT - Food Sci. Technol.* **2013**, *52*, 12–20, doi:https://doi.org/10.1016/j.lwt.2013.01.001.
99. Matwijczuk, A.; Oniszczuk, T.; Matwijczuk, A.; Chruściel, E.; Kocira, A.; Niemczynowicz, A.; Wójtowicz, A.; Combrzyński, M.; Wiącek, D. Use of FTIR Spectroscopy and Chemometrics with Respect to Storage Conditions of Moldavian Dragonhead Oil. *Sustainability* 2019, *11*.
100. Salimon, J.; Salih, N.; Abdullah, B.M. Improvement of Physicochemical Characteristics of Monoepoxide Linoleic Acid Ring Opening for Biolubricant Base Oil. *Biomed Res. Int.* **2011**, *2011*, 196565, doi:https://doi.org/10.1155/2011/196565.
101. Liu, W.; Xiao, B.; Wang, X.; Chen, J.; Yang, G. Solvent-Free Synthesis of Phytosterol Linoleic Acid Esters at Low Temperature. *RSC Adv.* **2021**, *11*, 10738–10746, doi:10.1039/D1RA00798J.
114. Atlaskina, M.E.; Atlaskin, A.A.; Kazarina, O. V; Petukhov, A.N.; Zarubin, D.M.; Nyuchev, A. V; Vorotyntsev, A. V; Vorotyntsev, I. V Synthesis and Comprehensive Study of Quaternary-Ammonium-Based Sorbents for Natural Gas Sweetening. *Environments* 2021, *8*.
115. Domańska, U.; Bogel-Łukasik, R. Physicochemical Properties and Solubility of Alkyl-(2-Hydroxyethyl)-Dimethylammonium Bromide. *J. Phys. Chem. B* **2005**, *109*, 12124–12132, doi:10.1021/jp058015c.
